# Progress and Recent Trends in the Application of Nanoparticles as Low Carbon Fuel Additives—A State of the Art Review

**DOI:** 10.3390/nano12091515

**Published:** 2022-04-29

**Authors:** Jeffrey Dankwa Ampah, Abdulfatah Abdu Yusuf, Ephraim Bonah Agyekum, Sandylove Afrane, Chao Jin, Haifeng Liu, Islam Md Rizwanul Fattah, Pau Loke Show, Mokhtar Shouran, Monier Habil, Salah Kamel

**Affiliations:** 1 School of Environmental Science and Engineering, Tianjin University, Tianjin 300072, China; jeffampah@live.com (J.D.A.); sandfran20@gmail.com (S.A.); jinchao@tju.edu.cn (C.J.); 2 Department of Mechanical and Automobile Engineering, Sharda University, Knowledge Park III, Greater Noida 201310, UP, India; abdulfatahabduyusuf@gmail.com; 3 Department of Nuclear and Renewable Energy, Ural Federal University Named after the First President of Russia Boris Yeltsin, 19 Mira Street, 620002 Ekaterinburg, Russia; agyekum@urfu.ru; 4 State Key Laboratory of Engines, Tianjin University, Tianjin 300072, China; 5 Centre for Green Technology, Faculty of Engineering and IT, University of Technology Sydney, Ultimo, NSW 2007, Australia; IslamMdRzwanul.Fattah@uts.edu.au; 6 Department of Mechanical Engineering, College of Engineering, Universiti Tenaga Nasional, Kajang 43000, Selangor Darul Ehsan, Malaysia; 7 Department of Chemical and Environmental Engineering, Faculty of Science and Engineering, University of Nottingham Malaysia, Jalan Broga, Semenyih 43500, Selangor Darul Ehsan, Malaysia; Pauloke.show@nottingham.edu.my; 8 Wolfson Centre for Magnetics, School of Engineering, Cardiff University, Cardiff CF24 3AA, UK; shouranma@cardiff.ac.uk; 9 Electrical Engineering Department, Faculty of Engineering, Aswan University, Aswan 81542, Egypt; skamel@aswu.edu.eg

**Keywords:** nanoparticles, biodiesel, vegetable oil, alcohol, research hotspots, fuel properties, engine characteristics

## Abstract

The first part of the current review highlights the evolutionary nuances and research hotspots in the field of nanoparticles in low carbon fuels. Our findings reveal that contribution to the field is largely driven by researchers from Asia, mainly India. Of the three biofuels under review, biodiesel seems to be well studied and developed, whereas studies regarding vegetable oils and alcohols remain relatively scarce. The second part also reviews the application of nanoparticles in biodiesel/vegetable oil/alcohol-based fuels holistically, emphasizing fuel properties and engine characteristics. The current review reveals that the overall characteristics of the low carbon fuel–diesel blends improve under the influence of nanoparticles during combustion in diesel engines. The most important aspect of nanoparticles is that they act as an oxygen buffer that provides additional oxygen molecules in the combustion chamber, promoting complete combustion and lowering unburnt emissions. Moreover, the nanoparticles used for these purposes exhibit excellent catalytic behaviour as a result of their high surface area-to-volume ratio—this leads to a reduction in exhaust pollutants and ensures an efficient and complete combustion. Beyond energy-based indicators, the exergy, economic, environmental, and sustainability aspects of the blends in diesel engines are discussed. It is observed that the performance of the diesel engine fuelled with low carbon fuels according to the second law of efficiency improves under the influence of the nano-additives. Our final part shows that despite the benefits of nanoparticles, humans and animals are under serious threats from the highly toxic nature of nanoparticles.

## 1. Introduction

The emergence of the industrial revolution coupled with modernization in lifestyle and vehicular population globally has led to a significant increase in energy demand, and according to Joshi et al. [1], global car ownership will double by the end of 2040 compared to 2016, by which conventional energy sources will power 80% of these cars. This trend has put excessive pressure on the global energy demand and supply market. Transportation uses 30% of the world’s total supplied energy, with road transport accounting for 80% of it. This sector is thought to account for approximately 60% of global oil demand and will continue to be the fastest expanding demand sector in the future [2]. Moreover, because of the increasing expansion of vehicles, demand for petroleum products is anticipated to climb to more than 240 million metric tonnes by 2021–2022 and to about 465 million metric tonnes by 2031–2032, assuming strong output growth [3]. The high energy demand from this market, together with other sectors, will eventually contribute to the exhaustion of the available petroleum reserves. In addition to resource depletion from excessive use of conventional fuels in the transport sector is their environmental emissions. Between 2007 and 2020, an estimated 4.1 billion metric tonnes of carbon dioxide were emitted into the atmosphere. Furthermore, between 2020 and 2035, an extra 8.6 billion metric tons of carbon dioxide is expected to be emitted into the environment [4,5]. For the aforementioned predicted timeframe, this is estimated to represent a 43 percent raise. Therefore, engine manufacturers are being forced to develop technologies and investigate cleaner alternative fuel sources without having to worry about engine changes due to strict pollution laws and rising energy demands.

Alcohols, biodiesel, and vegetable oils are among the most promising and popular liquid biofuels studied for their application in internal combustion engines (ICE). Though liquid biofuels such as biodiesels, vegetable oils, and primary alcohols have the potential of solving the world energy crisis, sometimes their direct application in conventional diesel engines is limited. For instance, biodiesels tend to oxidize quickly due to the presence of unsaturated fatty acids, which is the main disadvantage of biodiesel [6,7]. The presence of 11 wt% oxygen reduces its heating value compared to neat diesel fuel [8,9]. Vegetable oils are about 10–20 times more highly viscous [10,11]. The highly viscous nature of vegetable oils presents poor fuel atomization, negative cold flow characteristics, incomplete combustion, ring sticking, and carbon deposit in the combustion chamber, among many others [10,11]. The drawback with low carbon alcohols (i.e., methanol and ethanol) is that their combustion in diesel engines is characterized by lower efficiency as a result of their inherent inferior physico-chemical properties such as high latent heat of vaporization, low ignition qualities, and relatively poor calorific value [12,13]. Moreover, they are very hygroscopic, and thus, they have poor miscibility with diesel. These problems with the direct application of neat biofuels in engines are often circumvented by forming blends with diesel fuel. Many researchers acknowledge the fuel blending approach for achieving certain fuel characteristics in order to increase the performance and emission control of a diesel engine without modifying the present engine. Several researchers have focused on improving the quality of fuels with additives and emulsification [14,15,16,17]. With microscale additives, sedimentation, aggregation, and non-uniform size distribution are issues [18]. Particle sizes smaller than 100 nm may now be easily produced and utilized as additives in engines because of the progress made in nanoscience, resolving the aforementioned issues [19]. 

One of the most important and novel themes in ICE is nanotechnology. The extant literature in the field has shown that, under the influence of nanoparticles (NP), the aforementioned liquid biofuels and their blends with conventional fuels exhibit overall improved fuel properties and combustion characteristics. This significant improvement is as a result of the excellent thermophysical properties of NPs and their high reactivity characteristics, which are suitable for combustion in ICE. In addition, the high thermal conductivity of these NPs provides them with optimal heat and mass transport features [20,21,22,23]. For these NPs to be considered suitable additives for fuel combustion, Ribeiro et al. [24] outline the key requirements and features that should be present or exhibited by the NPs: (1) exhaust emissions should minimize after the addition of these additives to base fuels, (2) the presence of these additives should ensure that the oxygen concentration in the particle filter and the combustion chamber of the engine is boosted, (3) the stability of the nanofluids should not be a problem over a wide range of conditions, (4) the presence of the additives should see to it that the viscosity index of the resulting fuel blend is increased, (5) the additives should be able to produce an increased rate of ignition, i.e., the flash point and ignition delay period of the resulting fuel should reduce, and (6) wearing, friction loss, and corrosivity should not be a problem after introducing these additives to the base fuel.

The addition of nanoparticles to liquid fuels (biofuels and diesel) as a secondary energy carrier has enhanced combustion, performance, and emission properties. Numerous scientists have investigated the possibility of using these modified fuels in diesel engines. Several researchers have comprehensively reviewed the application of nano-additives in biofuel–diesel blends, including but not limited to Kegl et al. [25], Kumar et al. [18], Venkatesan et al. [26], Hoang [27], Shaafi et al. [28]; Khond and Kriplani [20], Dewangan et al. [29], Nanthagopal et al. [30], and Soudagar et al. [31]. Though these studies offer significant contributions to the corpus of literature, there exist some gaps that need to be filled;

(1) To the best of our knowledge, studies that holistically review all three biofuels (alcohols, biodiesel, and vegetable oil) in the context of nanoparticles and engine characteristics are scarce; most of these studies typically consider only one type of the biofuels, especially biodiesel, with limited review specifically dedicated for alcohols or vegetable oils in the broader spectrum.
(2) When doing a literature review on the evolution of any theory or concept over time, it is critical to include the development component by posing questions such as, “What are the evolutionary trends in the research field?”, “What future research areas have been emphasized in significant research articles?”, and “What are the major research areas?” [32]. The existing reviews clearly lack these aspects, and it is very imperative to systematically analyse the broad literature body, which could help structure the existing knowledge and identify future research gaps [33].
(3) Energy-based indicators of ICE such as brake thermal efficiency (BTE), brake specific fuel consumption (BSFC), and emission characteristics are usually the most used assessment criteria for nanofuels [34]. However, an assessment based on these energetic indicators alone is not enough to describe an all-round performance of the diesel engine [35]. In addition, it is difficult to examine the renewability and sustainability of an energy resource using energy analysis since this indicator fails to consider the effects of the second law’s limitation on energy conversion [36]. Exergy analysis bridges this gap as it is a combination of both first and second law of thermodynamics and is closely linked to the renewability and sustainability nexus. In order to achieve a better understanding of the irreversibility or resource destruction, one could employ exergy analysis as it is a powerful technique for investigating the imperfections in an energy conversion system [36,37]. Despite its tremendous ability to optimize energy systems, conventional exergy analysis is often criticized for overlooking the economics and environmental aspects of the thermal system being considered. In nutshell, for an overall performance of any fuel in a thermal system, the energy and exergy indicators are very important, but the addition of the economic and environmental analysis is also key in determining the profitability and sustainability of an improvement in process through exergo-economic and exergo-environmental analysis [35]. A number of studies on the aforementioned aspects related to nano-low carbon fuels in diesel engines have been conducted [34,35,36,38,39,40,41,42]—however, these generalized discussions are missing in the extant literature review papers on the current subject.
(4) It is worth noting that, besides the engine emissions, performance, and combustion characteristics, most of the existing reviews have only focused on the dispersion stability, wear and friction loss, corrosion, and cost-related issues with nanoparticles, with limited discussion on a very important aspect of these nano-additives, which is their toxicity and health impacts when they come into contact with humans and animals over a period of exposure. There is numerous evidence supporting how toxic these nanoparticles are and how detrimental they could be to an individual’s health [43,44,45,46,47,48]. It will therefore be prudent to augment the existing literature with these findings.

The review paper reports the impact of potential nanofuel additives on properties of fuel, engine performance, exhaust emissions, and combustion characteristics at different operating conditions. The past and present state of research of this field is also presented in the current work to reveal key research hotspots and ignored areas for future development. The exergy, economic, environmental, and sustainability of these nanofuels in low carbon fuelled-engines are reviewed. We conclude the current study with the toxicity and health impacts of nanoparticles based on results from literary sources.

## 2. Discussion on Zero Carbon Ecology and Circular Economy

Several organizations and experts around the world have emphasized that efforts to mitigate and adapt to climate change must be accelerated. Approximately 80% of the energy produced in the world comes from fossil fuels [49], with global fossil carbon emissions on the rise since the start of the last century. In this context, the transportation industry consumes approximately 21% of global energy, with oil accounting for 94% of that consumption and 8.0 Gt of direct carbon dioxide (CO_2_) emissions from fuel burning, accounting for almost a quarter of global totals [50,51]. This trend in emissions from the transportation sector has aroused significant attention from the scientific community in recent years as efforts are being made to attain a carbon neutral future.

Carbon neutrality, or achieving a carbon-free society, has piqued the interest of scholars, researchers, and policymakers throughout the last three decades. Countries around the globe are converting to renewable energy to reach carbon neutrality, with the goal of keeping global warming below 2 °C compared to pre-industrial levels [52,53]. To achieve a sustainable, low carbon, and resource efficient environment, modern concepts such the circular economy indeed have a significant role to play [54].

Recently, the principle of circular economy is gaining momentum in climate change mitigation measures, and it is believed to have an important role to play in reaching carbon neutral targets. Circular economy hinges on three main components, i.e., reduce, reuse, and recycle. In relation to carbon mitigation strategies particularly from major CO_2_ emitting sectors such as the transportation sector, circular economy can be translated to circular carbon economy through the use of alternative fuels in the following ways: (1) to reduce the carbon that must be managed in the first place, (2) to reuse carbon as an input to create feedstocks and fuels, (3) to recycle carbon through the natural carbon cycle with bioenergy, and (4) unique to circular carbon economy, to remove excess carbon and store it [55]. Based on the nature and characteristics of biofuels, they fit the bill in all four pathways of circular carbon economy. Hence, by increasing the share of biofuels in the transport sector, carbon emissions can be dramatically decreased, and chances of reaching carbon neutral targets are increased. In this context, nanoparticles indeed have a role to play in simultaneously promoting cleaner and efficient combustion of low carbon fuels in diesel engines.

## 3. Research Hotspots and Evolutionary Trends

Bibliometric analysis is one of the modern tools researchers have adopted in ascertaining the research focus and trend of a topic of interest. It is defined as applying mathematics and statistical methods to books and other media of communication [56]. It is a type of research study that provides the basis for what has been achieved and what needs to be investigated [57]. The methodology consists of descriptive and exploratory techniques deemed worthy tools to analyse the relevant literature’s recent trends [58]. Many researchers have used the tool to help identify research hotspots in different fields of science. To mention a few, in 2015, Mao et al. [59] conducted a bibliometric analysis on various renewable energy sources. Their study revealed that biogas and biodiesel were the two main areas researchers focused on between 1994 and 2013 regarding bioenergy. In another study, the same set of tools Min and Hao [60] employed between 1990 and 2017 to evaluate the research on biofuels; they found that the three most researched biofuels were biogas, vegetable oil, and bioethanol. Jin et al. [61] also comprehensively reviewed the past and current state of research on ethanol and methanol fuel combustion in ICE between 2000 and 2021. According to Zhang et al. [62], bibliometric investigation, Jatropha curcas, algae, waste cooking oil, and vegetable oil were the most hotspot-related papers for biofuel generation between 1991 and 2015.

As already mentioned in our introduction, to the best of our knowledge, the use of these tools in the area of nano-additives in liquid fuels for ICE does not exist in the literature. The ‘Web of Science’ Core Collection database was used to determine the historical and present research paradigms of the issue under consideration by utilizing the strategy and techniques of comparable bibliometric studies. Our search strategy is described as follows: “TOPIC: (nanoparticle* or nanoadditive* or ‘metal additive*’ or ‘nano emulsion’ or ‘nano material’) AND TOPIC: (‘diesel blend*’) Timespan: 2000–2021. The use of ‘diesel blend*’ is purposeful to reveal all related studies for diesel engine combustion. However, we also looked through the preliminary findings for publication titles and abstracts that were purely relevant to the present research. Thus, papers relating to any other biofuel either than biodiesel, alcohol, or vegetable oils were not included. Papers outside the scope of combustion, performance, and emission characteristics were also excluded. A total of 689 documents was finally retrieved and analysed with an R-statistical package (Biblioshiny) for identifying the core research focus for the subject of the current work.

**Figure 1:**
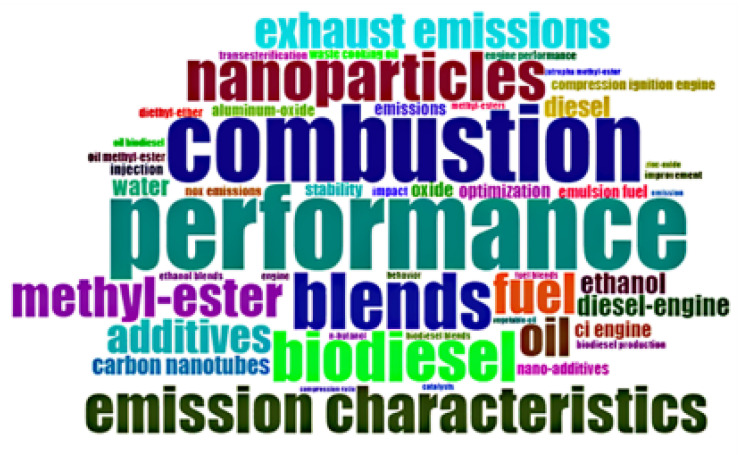
Top 50 keywords of research on nanoparticles as additives for biodiesel/vegetable oil/alcohol–diesel blends.

Figure 1 depicts the 50 most commonly used words or phrases in the subject area under consideration. PERFORMANCE, COMBUSTION, BLENDS, BIODIESEL, NANOPARTICLES, EMISSION CHARACTERISTICS, FUEL, and METHYL-ESTER are the words or phrases having at least 100 occurrences. These words suggest that the interest of the investigators in this research field lies in the application of nanoparticles as additives for improving the performance, combustion, and emission characteristics of liquid fuels. Besides these characteristics, STABILITY (rank 22) of liquid fuel–nano-additive blends is another area of interest in this field. One of the main concerns related to the application of NPs as additives for low carbon fuels is their stability aspects [31]. By virtue of their high surface activity and large surface, NPs are prone to aggregation-causing stability problems within the base fuel they are present in. Hence, more work is being carried out in this area to address the situation. Amongst the three biofuels under review in this work, biodiesel seems to be the most investigated fuel as far as nano-additive blending in liquid fuels is concerned. Other fuels in the top 50 keywords are ETHANOL, WASTE COOKING OIL, DI ETHYL ETHER, N-BUTANOL, and JATROPHA METHYL ESTER. Although ‘nanoparticles’ is a term representing several investigated nanoparticles or nano-additives, those to distinctively appear in the top 50 keywords were CARBON NANOTUBE, ALUMINIUM OXIDE, and ZINC OXIDE. It also appears that NOx emissions was the most frequently used environmental-related keyword.

**Figure 2:**
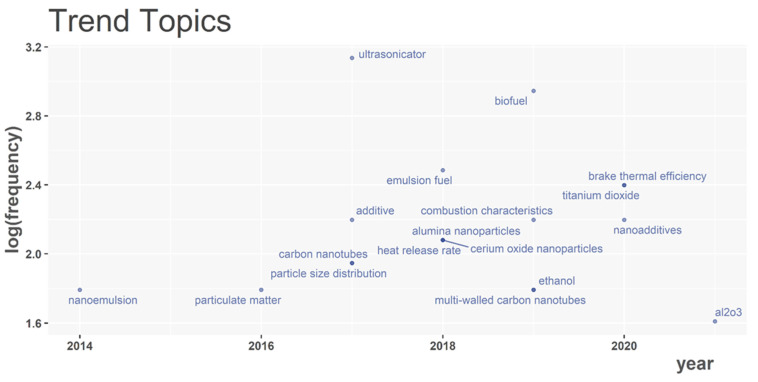
Analysis of topic trends for research on nanoparticles as additives for biodiesel/vegetable oil/alcohol–diesel blends between 2014 and 2021.

Following that, we add to the discourse by noting quantitative developments in nanoparticles as additives for biodiesel/vegetable oil/alcohol–diesel blends. Figure 2 shows a graphical representation of year-to-year research patterns. Prior to 2018, researchers were heavily involved in using CARBON NANOTUBES as additives mostly for controlling emissions, as seen in trend topics such as PARTICULATE MATTER, SOOT, and PARTICLE SIZE DISTRIBUTION. Some of the popular works completed in that period (according to citations) include: (1) Hosseini et al. [63], who studied the blends of carbon nanotubes and diesel-biodiesel and revealed that carbon monoxide (CO), unburned hydrocarbon (UHC), and soot emissions were dropped by 65.7, 44.98, and 29.41%, respectively; (2) Sadhik Basha and Anand [64], who used Jatropha biodiesel in the presence of carbon nanotubes to conduct their experiment. Their findings showed that smoke opacity and NOx emissions were 69% and 1282 ppm for the neat biodiesel, while the nano-emulsified fuel was 910 ppm and 49%, respectively; and (3) Heydari-Maleney et al. [65] analysed and investigated diesohol–B2 fuels under the influence of carbon nanotubes. Their results indicated that 6.69%, 31.72%, and 5.47% of soot, unburned hydrocarbons, and carbon monoxide were recorded, respectively. 

Furthermore, from the same Figure 2, it is seen that between 2018 and 2021, the attention shifted towards other nanoparticles such as CERIUM OXIDE (CeO_2_), TITANIUM DIOXIDE (TiO_2_), and ALUMINUM OXIDE (Al_2_O_3_). Kegl et al. [25] attempted to rank several nanoparticles and their base fuels under two main criteria; the first criteria considered Criterion A as representative of exhaust emissions and engine performance, whereas Criterion B, which was the second criterion, denoted only emission characteristics. Results from both criteria revealed that blends with Al_2_O_3_ delivered the most optimal feasibility for use in diesel engine. It is therefore not surprising that this nanoparticle has begun to attract the most interest in recent years, as seen in Figure 2.

**Figure 3:**
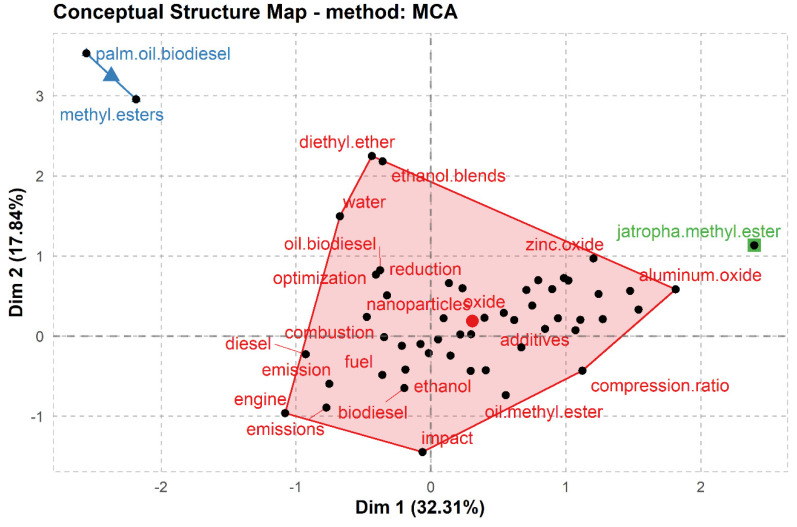
Conceptual structure plot using Multiple Correspondence Analysis (MCA).

In addition, the Multiple Correspondence Analysis (MCA) in R Biblioshiny was used to visualize the conceptual structure of the investigated topic. By using Porter’s stemming approach, this technique extracts terms from the papers’ title, keywords, and abstract—and in order to make these terms consistent throughout their usage, they are reduced to their base/root/word stem. Moreover, common themes can be identified by using K-means clustering technique to extract and group themes according to clusters. To conclude, the MCA takes into account the distribution of words according to their degree of similarity to construct a two-dimensional graphical map [58]. If two or more words focus on the same theme (common research theme), they are likely to appear closer to each other on the map away from unrelated themes. If a particular cluster is identified in the red region, then these themes have been paid attention to the most by the scientific community, while relatively less attention (relatively ignored) has been given to themes in green and blue clusters (Figure 3). The closer the dots on the graph representing each phrase are, the more similar the keyword distribution is, meaning that they co-occur more frequently in the articles. Furthermore, the proximity of a term to the centre point shows its importance in the study subject, whereas those at the margin are less relevant to other research topics.

The conceptual structure map aided in identifying the important research topics, their connections to other areas, and the topics that had attracted the least attention.

**Figure 4:**
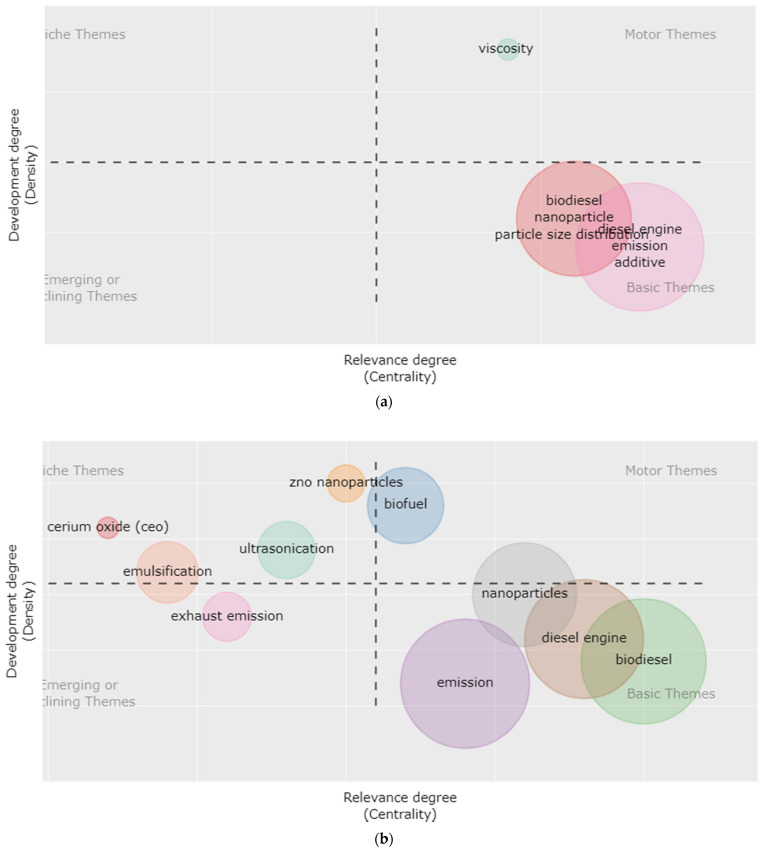
Thematic map of a research field from 2000 to 2010 (**a**); Thematic map of a research field from 2011 to 2021 (**b**).

The strategic diagram, as illustrated in Figure 4a,b, is a two-dimensional diagram that identifies two properties (“centrality” and “density”) that describe the themes. The degree to which one network interacts with another is measured by centrality. The centrality of a theme’s external ties to other subjects is measured and may be used as an indication to quantify the theme’s influence across the overall academic area. Density measures the strength of internal linkages among all keywords within a topic. As a result, the richness of a subject reflects its progression. The themes are divided into four quadrants based on their centrality and density. In the last decade, scholars have also improved their interpretation of this figure [66]; the interpretation is that, the first quadrant (central and developed) represents motor themes, the second quadrant (central and undeveloped) represents basic themes, the third quadrant (peripheral and developed) represents niche themes, and the fourth quadrant (peripheral and undeveloped) represents emerging or declining themes. Figure 4 is divided into two different periods; Figure 4a represents the themes of this research field during 2000–2010, whereas those of 2011–2021 is represented by Figure 4b.

The key themes with varied levels of density and centrality throughout the first half of the two decades (2000–2010) may be seen in the strategy diagram developed in Figure 4a, and they are VISCOSITY, BIODIESEL, NANOPARTICLE, PARTICLE SIZE DISTRIBUTION, DIESEL ENGINE, EMISSION, and ADDITIVE. Most of these themes were classified as important but undeveloped (according to their quadrant). However, since the Euro VI vehicle emission standard, Vehicular Emission Scheme, Bharat Stage IV, Paris Agreement, Sustainable Development Goals, and other global emission regulations came into effect in the last decade (2011–2021), several efforts have been made to make liquid fuel combustion cleaner and more efficient. Therefore, it is not surprising that the themes of this research field greatly intensified in the last decade, i.e., 2011–2021 (Figure 4b), compared to that of the first decade, i.e., 2000–2010 (Figure 4a). There has been a general increase in research interest in different nanoparticles and biofuels.

**Figure 5:**
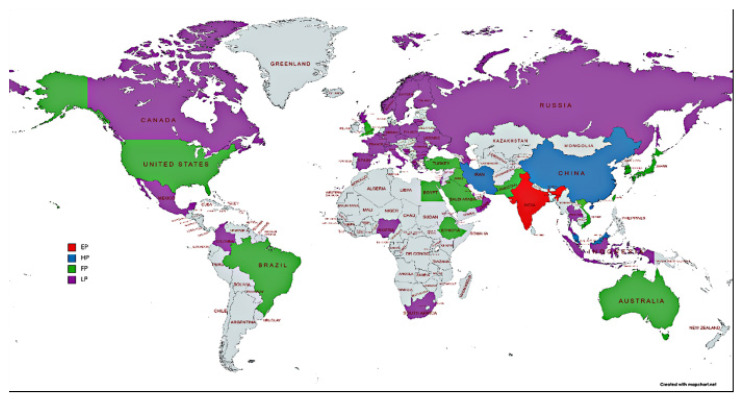
Geographical distribution of research on nanoparticles blending in alcohols/biodiesel/vegetable oil-based fuels (Note—EP: productivity is excellent; HP: productivity is high; FP: productivity is fair/average; LP: productivity is low).

Figure 5 shows the geographical distribution of the active researchers in this field of research. It can be seen that the field is largely driven by contributions from Asia, mainly by India (59.85%), China (9.57%), Malaysia (8.70%), and Iran (8.55%). Egypt (6.09%), Turkey (4.35%), USA (2.32%), and Brazil (1.45%) are the key contributors from Africa, Europe, North America, and South America, respectively. Studies from Africa, South America, and Oceania have been heavily underrepresented.

## 4. Fuel Properties, Emissions, Performance, and Combustion Characteristics

In this section, different fuel combinations from literary sources are reviewed, and the main results from these studies with respect to the effect of nanoparticles on the fuel properties, performance, emission, and combustion characteristics of diesel engines fuelled with alcohol/vegetable oil/biodiesel-based fuels are presented. 

Table 1 shows the elemental composition (carbon, oxygen, and hydrogen) of the base fuels considered in the current review. It is seen that for the low carbon fuels reviewed, the highest share of carbon is 77% while the lowest is 37.8%. Similarly, the hydrogen content and oxygen content ranges between 12 and 13.61% and 11 and 49.93%, respectively. The low carbon and oxygen content of these fuels relative to that of conventional diesel (87% carbon, 13% hydrogen, no oxygen) makes them cleaner for combustion in ICE. According to Low Carbon Technology Partnerships initiative (LCTPi), a low carbon fuel should have a CO_2_ performance significantly better than conventional fossil transport fuels by at least 50% [67].

The main fuel properties reviewed in this section are density, kinematic viscosity, cetane number, calorific value, and flash point. Under performance characteristics, we review evidence from the literature relating to brake specific fuel consumption (BSFC), brake thermal efficiency (BTE), brake torque (BT), and brake power (BP). However, there are currently limited experimental data on the brake torque and brake power after the inclusion of the nanoparticles for vegetable oils; thus, only BTE and BSFC are reviewed for this particular fuel. For combustion characteristics, we looked at in-cylinder pressure rise rate, ignition delay, and heat release rate (HRR). Finally, carbon monoxide, oxides of nitrogen, and hydrocarbon pollutants are reported under the emission characteristics.

### 4.1. Effect of Nanoparticles on Fuel Properties of Low-Carbon Fuels

**Figure 6:**
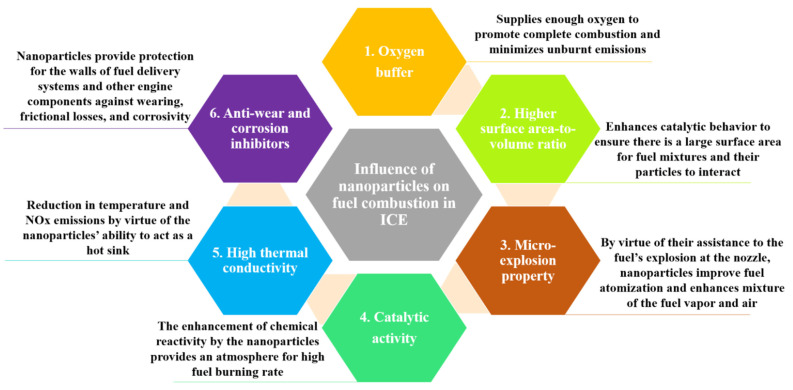
Characteristics of nanoparticles in CI Engines (Reprinted with permission from Ref. [30] Copyright © 2020 Elsevier).

The physico-chemical properties of a fuel tell how much influence it will have on the emission, performance, and combustion characteristics when fuelled in a diesel engine. For example, viscosity and density impact the duration of fuel atomization penetration; calorific value influences fuel consumption; and cetane number influences fuel ignition quality, resulting in more complete combustion. When these fuel properties are optimized, the resulting fuel provides better fuel performance, combustion, and emission characteristics. Nanoparticles have excellent characteristics, making them suitable as additives for various fuels. Nanthagopal et al. [30] summarized these excellent features of nanoparticles as shown in Figure 6. Several researchers have thus investigated the adjustment to fuel properties such as viscosity, density, flash point, cetane number/index, calorific value, etc., upon the addition of nanoparticles. Section 4.1.1, Section 4.1.2 and Section 4.1.3 are a summary of the effect of nanoparticles on fuel properties of alcohol, vegetable oil, and biodiesel-based fuels, respectively.

#### 4.1.1. Alcohol-Based Fuels

The inferior properties of alcohol-based fuels (especially low carbon alcohols, i.e., methanol and ethanol) such as poor ignition quality and lower heating value generally improve under the influence of nanoparticles. Carbon-based nanomaterials such as multi-walled nanocarbon tubes (MWCNT), graphene nanoplatelets [68], Fe_2_O_3_ [69], Al_2_O_3_ [70,71], and TiO_2_ [72] are generally better nanoparticle candidates for boosting the cetane number and calorific value of alcohol-based fuels. However, viscosity and density adjustments are dependent on the blend components. For example, zinc oxide (ZnO) [73], silicon dioxide (SiO_2_) [74], CeO_2_ [75] increases the densities of the original fuels. However, SiO_2_ [76], Fferric oxide (Fe_2_O_3_) [69], and Al_2_O_3_ [71] decrease the densities of the original fuels. The viscosity of the neat fuels worsens upon the addition of SiO_2_ [74], CeO_2_ [75], and TiO_2_ [72] but improves under the influence of the following nanoparticles; graphene oxide [77], multi-walled carbon nanotubes [68], graphene quantum dot [78], and Al_2_O_3_ [79]. Most of the authors added the nanoparticles in concentrations between 10 and 250 ppm (or mg/L). It is worth mentioning that the concentration of the added nanoparticles also had an effect of varying the fuel properties. For instance, when the concentration of SiO_2_ in neat methanol increases from 25 to 100 ppm, it negatively affects the density and viscosity of methanol [74]. Furthermore, when the concentration of Fe_2_O_3_ in N-amyl ternary fuel is increased from 40 to 120 ppm, the corresponding calorific value increases from 41.73 to 42.97 MJ/kg [69]. Table 1 summarizes the effect of various nanoparticles and their dosages on the properties of alcohol-based fuels from literary sources.

#### 4.1.2. Vegetable Oil-Based Fuels

The application of nanoparticles in vegetable oil-based fuels, to some extent, follows a similar pattern as that of alcohol-based fuels. Annamalai et al. [82] added cerium oxides in the concentration of 30 ppm to an emulsion of lemongrass oil. It was observed that the presence of the nanoparticle increased the densities and viscosities of the emulsion fuel. Similar observations have been made by Dhinesh et al. [83], where CeO_2_ was blended in Cymbopogon Flexuosu oil. With increase in the concentration of the nanoparticles, it was observed that the viscosity and density of the Cymbopogon Flexuosu oil became worse. Results from several works show that CeO_2_ may not be an ideal nanoparticle when the goal is to address the viscous and dense nature of vegetable oils [83,84,85,86,87]. On the other hand, Al_2_O_3_ had a positive effect on the density and viscosity of a pyrolyzed biomass oil when 50 ppm and 100 ppm of the nanoparticle was added to the base fuel [88]. Increasing the concentration of CeO_2_ in orange peel oil and lemon peel oil results in an increase in calorific value, but the trend reverses if CeO_2_ is replaced with carbon nanotubes [86]. Nano ferrocene shows excellent cetane-enhancing abilities in vegetable oil-based fuels than CeO_2_ [89]. Similarly, CeO_2_ also provides more energy content to oil-containing fuels than carbon nanotubes [90]. Some nanoparticles also had marginal or no effect on the properties of the based fuels. It is worth mentioning that, though nanoparticles can offer improvements to fuel properties, their concentrations in the blend should be moderated. Excessive addition of nanoparticles, especially CeO_2_, could defeat the original purpose of their inclusion in the base fuels. Table 2 summarizes the effect of various nanoparticles and their dosages on the properties of vegetable oil-based fuels from literary sources.

#### 4.1.3. Biodiesel-Based Fuels

El-Seesy et al. [92] blended multi-walled carbon nanotubes of concentrations between 10 and 50 mg/L into Jatropha biodiesel blends. The density of the original fuel remained unchanged, viscosity and cetane number increased, while a negligible difference was recorded in its calorific value. In a similar study, Alenezi et al. [93] increased the concentration of the multi-walled carbon nanotubes to 100 ppm in Palm oil biodiesel blends. The density and cetane number of the base fuel reduced, but its viscosity and calorific value increased. The density, viscosity, and calorific value of Jojoba biodiesel blends increased upon the addition of cupric oxide (CuO) (25–75 ppm) in the work of Rastogi et al. [94], but its flash point kept decreasing with an increase in the dosage of the nanoparticle. Al_2_O_3_ (0.2–0.04 ppm) has a positive effect on the density adjustment of Madhuca Indica, but it will decrease the methyl ester’s flash point and marginally/negligibly increase its calorific value, as shown by Rastogi et al. [95]. The addition of titanium oxide of 300 ppm to canola biodiesel produces a fuel that has improved density, viscosity, cetane number, and sulfur content than the neat biodiesel in the study of Nithya et al. [96]. Venu and Madhavan [80] show that Al_2_O_3_ (25 ppm) in Jatropha biodiesel-containing fuel will follow a similar trend observed in the work of Rastogi et al. [95] for density and viscosity, but assume an opposite trend in the flash point and calorific value. Janakiraman et al. [97] compared the fuel adjustment abilities of three different nanoparticles, namely TiO_2_, Zirconium dioxide (ZrO_2_), and CeO_2_, in Garcinia gummi-gutta methyl esters; in general, TiO_2_ and ZrO_2_ showed better fuel modification compared to CeO_2_. Following this observation, CeO_2_ generally offered negative fuel modifications when it was added to waste cooking oil according to Khalife et al. [98], but Karthikeyan et al. [99] stipulates otherwise; the addition of CeO_2_ to rice bran biodiesel had a positive effect on the density, viscosity, and flash point of the original fuel. Hajjari et al. [100] investigated the effect of CeO_2_ nanoparticles on the oxidative stability of biodiesel. Their results revealed that upon the addition of the nanoparticle at 50 ppm, the oxidative stability of the neat biodiesel worsened, but caused slight improvement when the concentration of CeO_2_ was increased to 200 ppm. However, at that high concentration, the oxidative stability of the resulting fuel still failed to meet the ASTM/EN requirement of 6 h induction period for biodiesel. Table 3 summarizes the effect of various nanoparticles and their dosages on properties of biodiesel-based fuels from literary sources.

### 4.2. Effect of Nanoparticles on Engine Performance/Emission/Combustion Characteristics of Low Carbon Fuels

#### 4.2.1. Engine Performance Characteristics of Nanoparticles in Alcohol-Based Fuels

##### Brake Thermal Efficiency

Prabakaran and Udhoji [73] revealed that the enhancement of surface area-to-volume ratio by zinc oxide nanoparticles led to an improvement in the BTE of diesel–ethanol–biodiesel blends at 100% load conditions and increased the average combustion temperature, as well as an exhibited an increase in the exhaust gas temperature along with the increase in load. Wei et al. [74] achieved a similar result for their blends of silicon dioxide nanoparticles and methanol. As the load increased, BTE increased, as well as being a result of the increased fuel injection quantity. Moreover, the increase in BTE became more obvious at higher concentrations of the silicon dioxide nanoparticle. In the study of Ramachander et al. [76], with a 40–120 ppm increase in silicon dioxide nanoparticles in a ternary fuel containing pentanol, the corresponding BTE increased by 1.58% to 2.34%. The authors explained that the catalytic activity of the nanoparticles may have resulted in finer combustion characteristics, thus positively influencing the BTE. Ağbulut et al. [70] revealed that the process of adding oxides of aluminum and titanium of 100 ppm concentration to diesel–bioethanol blends results in an increase of BTE of 5.70% and 5.15% for DF90E10 + A100 and DF90E10 + T100, respectively, compared to the DF90E10 test fuel. The increase in BTE of the base fuel upon the addition of the nanoparticles was attributed to the catalyst activity role thereof, micro exploits in primary droplets, the higher energy content of nanoparticles, their higher surface area to volume ratio, oxygen-buffer role, and superior thermal properties. According to Venu et al. [71], the addition of aluminum oxide nano-additives increased the BTE of ternary fuel (diesel–biodiesel–ethanol) by 2.48%, 7.8%, and 1.42% for doping concentrations of 10 ppm, 20 pm, and 30 ppm, respectively. The reasons behind this surge in BTE for the ternary fuel post doping were similar to those described in the work of Ağbulut et al. [70]. Another possible explanation was that the Al_2_O_3_ positively influenced the heat transfer rate due to its enhanced conductive, radiative, and heat mass transfer. The presence of Al_2_O_3_ ensured that the mixture of air with fuel vapor is enhanced, thereby promoting complete combustion.

##### Brake Specific Fuel Consumption

Shaafi and Velraj [79] added alumina nanoparticles (100 mg/L) to ethanol and iso-propanol as additives with diesel–soybean biodiesel blend (D80SBD15E4S1 + alumina fuel). A minimum BSFC was recorded for D80SBD15E4S1 + alumina fuel blend at 75 and 100% load conditions. The BSFC of B20, D80SBD15E4S1 + alumina fuel blend, and neat diesel was 0.312, 0.309, and 0.349 kWh, respectively. The large surface area of the alumina nanoparticles enhanced the combustion process of D80SBD15E4S1 + alumina fuel blend. El-Seesy and Hassan [72] shows that TiO_2_ nanoparticles’ presence in the Jatropha biodiesel–diesel–n-butanol blend (J50D10Bu) leads to a significant reduction in BSFC. The investigators explained that the high surface area of TiO_2_ nanoparticles resulted in a more reactive surface area with air, which improved evaporation rate and reduced ignition delay; thus, the combustion process enhanced. The presence of titanium dioxide nanoparticles in J50D10Bu reduced the BSFC up to 18% in contrast to that of the pure J50B10Bu blend. Diesohol fuel and B2 blends were doped with carbon nanotubes to investigate the effect on engine performance in the work of Heydari-Maleney et al. [65]. Carbon nanotubes were in concentrations of 20, 60, and 100 ppm. Results revealed that the addition of the carbon nanoparticle reduces BSFC of the base fuel. The addition of carbon nanotubes and ethanol in diesel fuel resulted in an average decrease in BSFC by 8.86%. The effect of alumina nano methanol fluid on the performance, combustion, and emission characteristics of a diesel engine fuelled with diesel methanol dual fuel was investigated by Zenghui et al. [112]. Three methanol-based nanofluids with Al_2_O_3_ of mass fractions 25, 50, and 100 ppm were prepared. Results from their investigation revealed that the methanol-based nanofluids recorded lower BSFC compared to the other test fuels without the nanoparticle. The lowest BSFC was recorded by the blend with the highest dosage of the nanoparticle (100 ppm). They claimed the accelerated evaporation mixing which led to an enhanced combustion process was a result of Al_2_O_3_ nanoparticles’ ability to decrease fuel droplet size and also positively influence the mixtures’ thermal conductivity. These processes eventually reduced the fuel requirement. In the work of El-Seesy and Hassan [77], the investigators reveal that by adding carbon nanomaterial, i.e., graphene oxide, multi-walled carbon nanotubes, and graphene nanoplatelets, to the blend of Jatropha biodiesel–butanol fuel (JME40B), a significant reduction in BSFC could be achieved. The carbon nanomaterial and the blends’ BSFC was reduced by approximately 35% compared to the pure JME40B. Some of the reasons the authors ascribed to this observation were that the reduction in consumed fuel and improved combustion process was as a result of an increase in the engine’s carbon oxidation rate by virtue of subjecting the JME40B to carbon nanomaterials doping. Moreover, these carbon nanomaterials shorten burnout time, which enhances the expansion work on the piston. As a result, a lower BSFC was obtained.

##### Brake Power and Brake Torque 

Örs et al. [81] performed a study to experiment with a ternary blend consisting of n-butanol, diesel, and biodiesel fuelled in a diesel engine under the influence of TiO_2_. The average BT of B20 + TiO_2_ increased around 10.20% compared to B20. Furthermore, the BT of B20But10 + TiO_2_ was approximately 9.74% higher than B20But10. The presence of n-butanol decreased the BT of the fuel blends but interestingly, doping the blend with TiO_2_ (B20But10 + TiO_2_) increased the BT value relative to B20. The corresponding maximum BP values at 2800 rpm were 9.17 kW by B20 + TiO_2_ and 8.59 kW for B20But10 + TiO_2_ in comparison with 8.16 kW for B20 and 7.69 kW for B20But10, respectively. The increase in BT and BP of the fuels after the addition of TiO_2_ was attributed to the high energy content of TiO_2,_ which is about 100–150 MJ/kg. In addition, the high surface–volume proportion of the TiO_2_ provided better oxidation of fuel; hence, high combustion enthalpy and energy density were released so that the maximum engine BT and BP increased. The exhaust emissions and engine performance of a single cylinder diesel engine fuelled with diesel–biodiesel–ethanol (DBE) ternary blend in the presence of nano-biochar was modelled by Mirbagheri et al. [113]. The average BT was remarkably increased by approximately 11.7% after the addition of the nano-biochar particles. The maximum BP of approximately 7.6 kW was recorded by DBE blends with a nano-biochar concentration of 113 ppm. According to the authors, the improved combustion process and atomization of the dispersed nano-organic particles resulted in an efficient conversion of the fuel blend’s chemical energy into mechanical work, which consequently boosted the BP and BT values. Diesohol–B2 blends were mixed with carbon nanotubes for evaluation on the characteristics of a diesel engine in the work of Heydari-Maleney et al. [65]. In this work, the fuel blends with carbon nanotubes produced the maximum BT while B2 and D100 produced the minimum BT. The increase in BT became very spontaneous by increasing the dosage of the carbon nanotubes. By doing this, the investigators claimed that the energy generated by the combustion in the cylinder is more complete, and the quality of combustion improves. Hence, the average pressure becomes greater, causing an increase in the piston force and torque. The results for BP are analogous to that of BT. The fuel blends with the carbon nanotubes recorded the highest BP, while B2 and D100 recorded the lowest BP. At higher concentrations of the carbon nanotubes, combustion improves and energy conversion to useful work becomes more effective. This observation could be due to the increase in heat transfer co-efficient attributed to the carbon nanotubes’ high surface area-to-volume ratio. Ethanol–biodiesel blends doped with Graphene Quantum Dot (GQD) nanomaterials were researched by Heidari-Maleni et al. [78]. By virtue of GQD’s influence, the brake power and brake torque of the oxygenated blends increased by 28.18% and 12.42%, respectively. Additionally, according to Safieddin Ardebili et al. [114], the presence of nano-biochar (SNB) slightly increased the brake power of fusel oil–diesel fuel by ~3%. The researchers explained that the catalytic activity of the SNB particles contributed to reducing ignition delay, which resulted in higher peak cylinder pressure and a better combustion process. At 100 ppm, full load condition, and 2000 rpm engine speed, the highest BT value of 7.8 Nm was recorded for 10% fusel oil. When the test conditions were kept constant, the corresponding engine torque was approximately 7.4% without the SNB particles.

#### 4.2.2. Engine Emission Characteristics of Nanoparticles in Alcohol-Based Fuels

##### Carbon Monoxide

Mardi K. et al. [115] created three nano emulsions fuel, namely, BD.CNT.DEE.E, BD.ALO.EHN.M, and BD.TIO.GLC.B, with nanoparticles of CNT, Al_2_O_3_, and TiO_2_, respectively of concentration 50 ppm. BD.CNT.DEE.E showed the lowest CO emissions compared with the other emulsion fuels and a 26% decrease from biodiesel values, whereas BD.TIO.GLC.B and BD.ALO.EHN.M showed 20% and 12% CO reduction, respectively. CNT has better combustion attributes and higher oxygen content of ethanol, improved fuel atomization of DEE, and better formation of air–fuel mixture by micro-explosions of water led to complete oxidation of the fuel mixture, and hence, the highest reduction in CO emissions for the BD.CNT.DEE.E emulsion fuel. In the work of Soudagar et al. [116], biodiesel was blended with octanol under the influence of 3% of functionalized MWCNTs. The authors claimed that MWCNT nanoparticles were inefficient for promoting combustion. The CO emission of MWCNT-containing fuels increased by an average of 38.4% more than diesel at all loads. According to Venu et al. [71], the presence of 10–30 ppm of alumina nanoparticles in a ternary fuel made up of diesel–biodiesel–ethanol reduces CO emissions by 2.81–11.24% compared to the neat ternary fuels. According to the authors, alumina nanoparticles have the ability to act as an oxygen donating catalyst and buffer for CO molecules’ oxidation. In addition, the chemical reactivity enhances leading to a decrease in the ignition delay period by virtue of the nanoparticle’s large surface area-to-volume ratio. These processes promote complete combustion and reduce emissions of CO. It is worth mentioning that Al_2_O_3_ nanoparticles dissociate to Al_2_O and O at elevated temperatures. Inside the combustion chamber, ‘Al_2_O’ is very unstable at those extreme temperatures, and this further decomposes it to 2Al and $\frac{1}{2}$O_2_. As seen in Equations (1)–(3), CO_2_ is produced from further reaction of this oxygen molecule with the CO. The above-mentioned mechanism contributes to a much-lowered CO emission.

Al_2_O_3_ → Al_2_O + 2O (1)

(2) $${\mathrm{Al}}_{2}O\;\to \;2\mathrm{Al}+\frac{1}{2}O_{2}\;$$

O + CO → CO_2_ (3)

According to El-Seesy and Hassan [72], the presence of titanium dioxide in the blend of Jatropha biodiesel–diesel–n-butanol blends ensured a significant decrease in CO emissions. The reduced ignition delay period and ignition characteristics enhancement by the action of the TiO_2_ nanoparticles was responsible for this observation. Moreover, these nanoparticles improve fuel–air mixing inside the combustion chamber as a result of their high catalytic activity—and the process aids in the reduction of CO emissions. Nutakki et al. [69] prepared a blend of n-amyl alcohol/biodiesel/diesel blend with(out) the influence of iron oxide nanoparticles, whose dosages were 40 ppm (TF40), 80 ppm (TF80), and 120 ppm (TF120). The CO emissions in TF40, TF80, and TF120 were 7.89%, 11.23%, and 23.26% lower than the ternary fuels without the nanoparticles. The researchers supported their results with the explanation that iron oxide nanoparticles are inherently high oxygen-bearing in nature, which aids in the oxidation of CO molecules as a result of the nanoparticles’ high catalytic activity. The reduction in CO emissions, according to the authors, is also attributed to the improved combustion process due to the high surface area per volume of the nanoparticles, causing ignition delay period to shorten.

##### Hydrocarbons

Pan et al. [75] investigated the impacts of adding cerium dioxide nanoparticles to methanol on the combustion, performance, and emission of a dual-fuel diesel engine. Without the CeO_2_, HC emissions in methanol mode increased significantly, particularly for the cases with higher methanol concentrations. However, with the addition of CeO_2,_ HC emissions are effectively reduced irrespective of the operating conditions. Compared to methanol mode, the maximum reduction in HC emissions were 47.8%, 56.3%, 31%, and 41.1% for M10Ce25, M10Ce100, M30Ce25, and M30Ce100, respectively. The authors provided explanations for the trend in HC emissions of methanol obtained under the influence of CeO_2_: (1) CeO_2_ nanoparticles as oxygen buffers provide oxygen atoms to improve combustion and hence reduce HC emissions. (2) CeO_2_ exists as an oxidation catalyst that can promote the oxidation of hydrocarbons in which CeO_2_ is converted to Ce_2_O_3_, according to Equation (4). (3) The catalytic activity of CeO_2_ nanoparticles can lower the combustion activation temperature of carbon and promote more complete combustion.

(4) $$(2X+Y)\;{\mathrm{CeO}}_{2}+{\mathrm{CxH}}_{y}\;\to \;(\frac{\left(2X+Y\right)}{2})\;{\mathrm{Ce}}_{2}O_{3}+\frac{X}{2}\;{\mathrm{CO}}_{2}+\frac{Y}{2}\;H_{2}O$$

Silicon dioxide nanoparticles were blended as additives to methanol in the work of Wei et al. [74]. Their results revealed that the increase in the concentration of the SiO_2_ resulted in a significant reduction in HC emissions regardless of the engine loads and methanol substitution ratios. The catalytic action of the SiO_2_ nanoparticles may have played a role in the HC reduction by lowering the combustion activation temperature of carbon to promote combustion. Furthermore, SiO_2_ nanoparticles provided additional oxygen molecules to help promote combustion. A maximum reduction in HC emission of 74.2 could be possible due to the presence of the SiO_2_over the tested conditions. Heidari-Maleni et al. [78] experimented on an ethanol–biodiesel blend using graphene quantum dot (GQD) nanoparticles. Due to the high catalytic activity of the nanoparticles, their surface area-to-volume ratio increases and thus produces more energy inside the cylinder to obtain more complete fuel combustion and reduce emission of pollutants. By adding GQD to ethanol–biodiesel blends, HC emissions reduced by 33.12%. Three different nanoparticles viz graphene oxide (GO), graphene nanoplatelets (GNP), and multi-walled carbon nanotubes (MWCNT) were added as fuel additives to n-butanol–Jatropha biodiesel by El-Seesy and Hassan [77] to investigate its performance on a diesel engine. Under the influence of the nanoparticles, the HC emissions of JME40B were significantly reduced. These nanoparticles can shorten ignition delay and improve ignition characteristics of the fuel they are blended into. Moreover, because these nanoparticles have a higher surface area to volume ratio, they exhibit high catalytic activity, and this attribute helps promote fuel–air mixing during the combustion process. The above-mentioned factors may be the reason for the nanoparticles’ positive impact on the HC emissions according to the researchers. The results from their work showed that an approximately 50% reduction in HC emissions could be achieved for the blends of the JME40B + nanoparticles. Venu and Madhavan [80] compared two different additives for biodiesel–diesel–ethanol blends, i.e., diethyl ether and alumina nanoparticles, for their combustion, performance, and emission characteristics. Their results showed that the blends with diethyl ether recorded more unburned HC. However, the alumina-containing blends exhibited lower HC emissions throughout the engine load except at full load conditions. The catalytic combustion activity of Al_2_O_3_ was well recognized for lower and part loads and may have improved the combustion process, thereby lowering the HC emissions.

##### Nitrogen Oxides

Khan et al. [117] prepared a nanofluid involving Nigella Sativa biodiesel, diesel, n-butanol, and graphene oxide nanoparticles with the aim of enhancing the performance, combustion, and symmetric characteristics and reducing the emissions from a diesel engine. They concluded that NO_x_ emissions from the nanofluid were higher than that of neat diesel and the diesel–biodiesel blends. Per the explanations given by the researchers, the presence of the graphene oxide added more oxygen molecules to what n-butanol and biodiesel had already added. The excess oxygen molecules in the nanofluid may have contributed to higher NO_x_ emissions. Mehregan and Moghiman [118] numerically investigated the effect of nano aluminum on NO_x_ and CO pollutants emission in liquid fuels combustion. Their analysis revealed that the mass fraction of NO_x_ pollutants decreases by adding the aluminum nanoparticles to ethanol and n-decane liquid fuels. Their results confirmed that aluminum nanoparticles, due to their enhanced thermal conductivity, led to improved combustion features of ethanol and n-decane liquid fuels. El-Seesy et al. [68] showed that the addition of carbon nanomaterials (multi-walled carbon nanotubes, graphene oxide, and graphene nanoplatelets) to a blend of n-heptanol–diesel leads to an increase in NO_x_ level at various engine loads, except at high loads. El-Seesy and his team explained that at lower and part loads, the increase in NO_x_ emissions is attributable to the positive effect of the carbon nanomaterials and n-heptanol that lead to an increase in peak pressure increases NO_x_ emissions (Zeldovich Mechanism). However, at higher loads, the presence of the additives may have reduced combustion duration; therefore, there was not sufficient time for the formation of NO_x_. By adding aluminum oxide and titanium oxide to diesel–bioethanol blends, Ağbulut et al. [70] showed that the process resulted in a 6.40% and 4.99% drop in NO_x_ emission for DF90E10 + A100 and DF90E10 + T100, respectively, compared to neat DF90E10. The investigators explained that the main reason behind this drop in NO_x_ emission might be due to the increase in thermal conductivity of the blends after the addition of the nanoparticles, which ensured a rapid heat transfer for the resulting fuel. Thus, the proper elevated temperature required for the formation of NO_x_ was not highly reached, and NO_x_ emission was seen lesser with the nanoparticle-doped fuels. In the work of Nour et al. [119], Al_2_O_3_ was added to diesterol (70% diesel+ 20% ethanol+ 10% Jojoba biodiesel) blends. Without the nanoparticles, JE20D exhibited a higher NO_x_ emission especially at lower loads. The addition of the Al_2_O_3_ nanoparticles to JE20D caused no impact in NO_x_ emissions at lower loads. However, at high engine loads, lower NO_x_ was reported for JE20D25A, JE20D75A, and JE20D100A in comparison to pure diesel and JE20D blends. The authors ascribed this trend to the high catalytic behaviour of Al_2_O_3_ nanoparticles that led to a more complete combustion, forming the final products with a minimum thermal breakdown of the hydrocarbon compounds. Hence, per the existence of lower active radicals, the possibility of forming thermal NO_x_ was lowered. 

#### 4.2.3. Effect of Nano-Additives and Diesel–Alcohol Fuels on Engine Combustion

The adverse effect of diesel fuel usage in CI engines has created significant interest in prospective renewable additives, such as alcohol, including butanol, ethanol, and methanol. These fuels can be used as emulsion, dual, or blend with diesel and biodiesel, to enhance fuel properties and stability. Small changes in combustion might not yield a significant improvement in cylinder chamber. However, it is necessary to create the correct mixture conditions, in particular, to control the air movement and turbulence [120]. Table 4 summarizes the recent experiments on the variation in combustion characteristics from CI engines fuelled with various nano-additives and alcohol fuels.

The effect of Al_2_O_3_ NPs with a dosing range of 10 ppm–30 ppm in ethanol fuel on combustion characteristics was investigated by Venu et al. [71]. It was found that the in-cylinder pressure decreases by 2.33% with TF20 as compared to diesel fuel. This trend absolutely matches with results found by Ağbulut et al. [70] with TiO_2_ and Wei et al. [121] with Al_2_O_3_, they observed a significant reduction in the peak cylinder pressure for ethanol and methanol fuels due to the high specific heat of alcohol fuels and high latent heat of vaporization, which could be reason for the drop-in peak [121]. On the contrary, the in-cylinder pressure significantly improved with the presence of CeO_2_ [75], Fe_2_O_3_ [69], and ZnO [73] nano-additives with different alcohol-based fuels. This is linked to the shortened ignition delay using nanofluids due to high thermal conductivity and surface area to volume ratio. 

El-Seesy and Hassan [72], Örs et al. [81], and Yaşar et al. [122] assessed the impact of butanol with nanoparticles TiO_2_ as a diesel engine catalyst. They discovered that the fuel with 25 ppm and 50 ppm TiO_2_ NPs produced superior combustion efficiency with better emission reduction as compared to diesel–butanol fuels without TiO_2_. Heidari-Maleni et al. [78] found that when GQD NPs concentration is elevated (added to ethanol fuel), the peak HRR is reduced by ~14.35% compared to that of diesel, consequently demonstrating a less combustible mixture formed at low cylinder temperature. Similar evidence in reduction with nano-biochar/diesel–ethanol was observed using a dosing range of 25 ppm–125 ppm [113]. For diesel–methanol fuel, the maximum peak HRR is obtained at 100 ppm dosage of nano-additives with up to 7.79% increase with CeO_2_ [75] and 8.6% increase with SiO_2_ [74], respectively.

#### 4.2.4. Engine Performance Characteristics of Nanoparticles in Vegetable Oil-Based Fuels 

##### Brake Thermal Efficiency

Polanga oil-diesel blends were doped with iron oxide nanoparticles for evaluation on a CI engine by Santhanamuthu et al. [91]. It was seen that the BTE of the blends with iron oxide nanoparticles was on par with that of neat diesel. According to the authors, the improvement in BTE due to the presence of iron oxide can be attributed to the enhancement of thermal properties such as thermal conductivity, thermal diffusivity, and convective heat transfer co-efficient that the nanoparticles present. Purushothaman et al. [125] added 25–100 ppm of Al_2_O_3_ and TiO_2_ nanoparticles alternatively to mahua oil. It was reported in this work that the BTE value of 100 ppm Al_2_O_3_ and TiO_2_ blended emulsified mahua oil were 29.2% and 28.4%, respectively, compared to 23.8% of neat mahua oil. From the authors’ perspective, the nanoparticles acted as a heat source which shortened the ignition delay and also enhanced the combustion due to the higher surface area to volume ratio. In the work of Ramesh et al. [126], canola oil blended diesel with Al_2_O_3_ nanoparticles was optimized through single and multi-objective optimization techniques. Results from their experiment showed that 18.8% of canola blends with 30 ppm of nanoparticles had BTE of 33.81%, which was a 16% increase with reference to pure diesel. Additionally, 10–30 ppm of alumina nanoparticle was added to lemongrass oil by Balasubramanian et al. [127]. The BTE of B20A20 was higher than any other test fuel at low and medium engine load conditions. At medium load, BTE of B20A20 increased by 12.24% and 4.08% over B20 blend and neat diesel, respectively. Moreover, at 100% load, BTE of B20A20 increased by 2.71% over B20 fuel. The authors mentioned that the presence of the alumina nanoparticle provided more oxygen molecules that boosted the combustion inside the cylinder. This was possible due to a higher area to volume ratio, improved atomization, quick evaporation, and greater mixing of fuel and air brought about by the alumina nanoparticle. Dhinesh et al. [83] showed that the addition of cerium oxide (10–30 ppm) to Cymbopogon flexuous biofuel was blended with diesel fuel positively impacts BTE. C20-D80 + 20 ppm CeO_2_ resulted in higher BTE than C20-D80 blend. The authors explained that the presence of CeO_2_ in the base fuels acted as a catalyst and oxygen buffer for combustion enhancement.

##### Brake Specific Fuel Consumption

The impact of rice husk nanoparticles on a diesel engine running on pine oil–diesel blends was investigated by Panithasan et al. [128]. It appears that the pine oil blends with 0.1% rice husk nanoparticles consume less fuel than the blends without nanoparticles. They explained that the rice husk nanoparticle acted as an oxygenated additive which enhanced the combustion process. Sathiyamoorthi et al. [85] studied the combined effect of nano emulsion and EGR on the characteristics of neat lemongrass oil–diethyl ether–diesel blend. BSFC of the cerium oxide-based nano emulsified LGO25 with EGR mode was increased by 10.8% compared to LGO25. They attributed this rise in BSFC to the lower calorific value of the nano emulsified LGO25, although the high cetane number and oxygen content of diethyl ether could partially reduce BSFC while operating in EGR mode. According to Dhinesh and Annamalai [87], utilizing cerium oxide nanoparticles mixed with an emulsion of Nerium oleander biofuel results in lower energy consumption when compared to neat Nerium oleander. The energy consumption of the nano emulsified fuel was 13.33 MJ/kWh whereas the neat Nerium oleander was 14.21 MJ/kWh. In a similar study, Annamalai et al. [82] dispersed 30 ppm of ceria nanoparticles into lemongrass oil (LGO) emulsion fuel. The process resulted in a nano emulsified fuel with energy consumption of 12.99 MJ/kWh, whereas that of neat LGO and diesel were both 13.8 MJ/kWh. In both studies, the researchers supported this observation claiming that, by introducing cerium oxide to the emulsion of Nerium oleander biofuel/lemongrass oil, the secondary atomization and micro-explosion improved, which in turn resulted in a heightened evaporation rate and mixing of the fuel. In the study by Dhinesh et al. [83] involving *Cymbopogon flexuous* biofuel was blended with diesel fuel under the influence of cerium oxide nanoparticles, lower energy consumption was achieved in the nano-blended fuels than the non-nano blended fuels. The researchers attributed cerium oxide’s catalytic ability and oxygen buffer which promotes complete combustion as the reason behind the obtained results.

#### 4.2.5. Engine Emission Characteristics of Nanoparticles in Vegetable Oil-Based Fuel

##### Carbon Monoxide

Panithasan et al. [128] experimented with rice husk nanoparticles as additives for pine oil–diesel blend. Results showed that at full load condition, CO decreases by about 27.27% more than diesel fuel. According to the authors, the oxygen content in both rice husk nanoparticles and pine oil increased the combustion rate and aided the addition of CO into CO_2_. Chinnasamy et al. [88] reported that by adding 50 ppm of Al_2_O_3_ nanoparticles into pyrolyzed biomass oil, there was a reduction in CO emissions compared to that of neat diesel and pyrolyzed biomass oil. From the authors, the presence of the nanoparticles improved the ignition characteristics and led to high catalytic activity. This is due to the higher surface area to volume ratio of the Al_2_O_3_, which resulted in an enhanced air-fuel mixing in the combustion chamber which further reduced the CO emissions. According to Sheriff et al. [86], 50 ppm of cerium oxide in lemon peel oil–diesel and orange peel oil–diesel resulted in percentage values for CO emissions of 0.223% and 0.092%, respectively, at full load; in a similar manner, that of 50 ppm of carbon nanotubes in lemon peel oil–diesel and orange peel oil–diesel were both 0.225%. At the same load, the CO emissions for neat diesel was 0.251%. By blending two different nanoparticles in mahua oil fuel, Purushothaman et al. [125] showed that Al_2_O_3_ had better CO emission reduction than TiO_2_. This was as a result of Al_2_O_3_ higher thermal conductivity than TiO_2_. Sathiyamoorthu et al. [85] provided evidence to the fact that the addition of cerium oxide to emulsified LGO25 decreased the base fuel’s CO emission by 7.14% and 4.87% when compared to LGO25. From the investigators, the presence of cerium oxide shortened ignition delay with better fuel–air mixing that could have led to the uniform burning process in the combustion chamber and promoted more complete combustion.

##### Hydrocarbon

In the study of Purushothaman et al. [125], HC emissions from emulsified mahua oil with Al_2_O_3_ and TiO_2_ were significantly lowered compared to other neat test fuels. The HC emission values of 100 ppm Al_2_O_3_ and TiO_2_ nanoparticle-blended EMO were found to be 57 ppm and 61 ppm, respectively, in contrast to 65 ppm and 91 ppm for diesel and mahua oil. The combined effect of micro explosion and high in-cylinder temperature due to the nanoparticles may have contributed to this reduction in HC emissions. Elumalai et al. [129] experimented on harmful pollution reduction technique in a low heat rejection (LHR) engine fuelled with blends of pre-heated linseed oil and TiO_2_. The blends with nanoparticles had lower HC emissions than the other base fuels without nanoparticles. The HC emissions of blends PLSNP50, PLSNP100, PLSNP150, PLSNP200 are −7.35%, −22.10%, −29.41%, and −33.82%, respectively, compared with the PLS20 in LHR engine. From their work, the authors explained that the addition of nanoparticles to preheated fuel led to a rapid burning of fuel due to the oxygen influx from TiO_2_ and minimized the carbon content during combustion. With cerium oxide acting as an oxidizing agent, Dhinesh and Annamalai [87] showed that NENOB (emulsion with nanoparticle) provided a 20%, 30%, and 36.3% reduction in HC emissions when compared to the other fuels without the nanoparticles (NOB, ENOB, and SFDF, respectively). Furthermore, hydrocarbons react with cerium oxide to form various products such as CO_2_, water, and cerous oxide, thereby limiting HC emissions. Panithasan et al. [128] showed that rice husk nanoparticles in the blend of pine oil–diesel (B20-0.1%RH) at full load conditions decreases the HC emissions by 19.64% compared to neat diesel. The researchers explained that the excess oxygen molecules delivered by the nanoparticle prevented the hydrocarbons from escaping the combustion process, thereby reducing the HC emissions from the exhaust gases. In the work of Sheriff et al. [86], it was revealed that 50 ppm CNT nanoparticle in lemon peel oil blend showed relatively less HC emission than that of cerium oxide due to the higher surface to volume ratio of the former, which led to improved air–fuel mixing. However, the trend reverses when lemon peel oil is exchanged with orange peel oil.

##### Nitrogen Oxides

In the presence of iron oxide nanoparticles, NO_x_ emission was reduced to 50% of that of neat diesel at higher Polanga oil, according to Santhanamuthu et al. [91]. The reason was that the iron oxide acted as a catalyst for the reaction of the hydroxyl radicals present in the Polanga oil and lowered the oxidation temperature. Balasubramanian et al. [127] presented results for their investigation on a diesel engine fuelled with lemongrass oil and alumina nanoparticles. The results showed that the addition of the nanoparticle lowered the NO_x_ emissions of the base fuels. At medium and high load conditions, there was a 19.23% and 1.73% increase in NO_x_ emissions for B20 blend and neat diesel. These values corresponded to a significant decrease in NO_x_ emission values for the B20A20 blend of 1.53% and 2.25% at medium and higher load conditions, respectively. They gave reasons that the alumina nanoparticles acted as a reducing agent and an oxygen absorber to reduce the NO_x_ emissions. Ceria nanoparticles of dosage 30 ppm were blended in emulsion fuel of lemongrass oil for assessment on performance, combustion, and emission characteristics in the work of Annamalai et al. [82]. It was revealed that the NO_x_ emissions of LGO nanoemulsion reduced by 24.8% and 20.3% compared with LGO and diesel fuels, respectively. According to the authors, the nanoparticle acted as a reduction agent. The oxides of nitrogen are reduced to form nitrogen and oxygen as a result of the high thermal stability of cerous oxide formed from the oxidation of unburned hydrocarbon. The soot remained stable and active after enhancing the initial combustion cycle, which may have significantly reduced NO_x_ emission. Equation (5) represents the chemical reaction described above.

(5) $${\mathrm{Ce}}_{2}O_{3}+\mathrm{NO}\;\to \;2{\mathrm{CeO}}_{2}+\frac{1}{2}N_{2}$$

Unlike the case of CO emissions, the higher thermal conductivity of Al_2_O_3_ became a disadvantage in terms of NO_x_ emissions when compared to TiO_2_ as nano-additives for mahua oil [125]. The NO values of 100 ppm Al_2_O_3_ and TiO_2_ nanoparticle-blended EMO in the work of Purushothaman et al. [125] were found to be 260 ppm and 275 ppm, respectively, whereas, for diesel and mahua oil, the respective values were 537 ppm and 289 ppm. According to Panithasan et al. [128], the addition of rice husk nanoparticles to diesel-pine oil blend had a negative impact on NO_x_ emissions; at full load conditions, the NO_x_ emission of B20-0.1%RH increased by 8.76% compared to neat diesel. The authors attributed this observation to the additional oxygen content provided by the nanoparticles, which caused an increase in the in-cylinder temperature of the combustion chamber and thereby assisted in increasing the NO_x_ level. 

#### 4.2.6. Effect of Nano-Additives and Diesel–Vegetable Oil Blend Fuel on Engine Combustion

Table 5 summarizes the most recent experiments on CI engines fuelled with various nano-additions and vegetable-based oils. Despite the benefits associated with bio-oil/diesel blends in CI engines, the usage of bio-oil as blend fuel gives a few drawbacks, such as large variation in fuel consumption [129], less calorific value and density [130], and decrement in mileage on vitality premise by ~10% [84]. To overcome these drawbacks, it is often necessary to improve it with suitable nano-additives and by appropriate combustion management. However, distinct species of nano-additives, such as Al_2_O_3_, CeO_2_, MgO, rice husk NPs, SiO_2_, MWCNTs, and TiO_2_ in bio-oils, are used to obtain better fuel properties over a long period of time [25,131,132], and may improve the engine combustion [86].

Among other research investigated, Balasubramanian et al. [127], Chinnasamy et al. [133], and Purushothaman et al. [125] examined the effect of Al_2_O_3_ NPs with bio-oil based fuels from the diesel engine, and a significant increase was observed with heat release rate and in-cylinder pressure leading to an increase in thermal efficiency. They attributed these results to a rise in ignition delay, and combustion duration causes the in-cylinder soot to be more highly oxidized, hence promoting the oxidation rate of soot particles which is then higher than the specific active surface rate [134,135,136]. Similarly, the diffusive combustion phase is shortened due to the addition of CeO_2_ NPs into waste pyrolysis and orange oils, which raises the range of ignition delay that helps in accelerating the combustion [86,137]. 

Although other researchers investigated that increasing the concentration of nano-additives such as CeO_2_, Ce_0.7_Zr_0.3_O_2_, and MgO in bio-oil affects the peak heat rate, in-cylinder, and peak pressure due to the higher energy droplet aggregation during spray atomization [82,87,138,139] later resulted in high fuel consumption [30]. That means not all nano-additives and bio-oil fuels contribute to the in-cylinder chamber; assessment needs to be made for notable nanofluids selected. Besides, a rise in the concentration of TiO_2_ and MWCNTs with vegetable-based fuels resulted in a higher heat release rate and cylinder pressure [140,141]. Furthermore, the presence of water molecules in emulsion fuel and vegetable-oil fuels leads to an increase in ignition delay and in-cylinder pressure, which suddenly favours heat release rate [106,142]. However, most of the literature reported that the addition of nano-additives facilitated the uniform distribution and stable suspension of fuel in the combustion chamber, resulting in an increase in the penetration length of the spray [25,27,143]. 

#### 4.2.7. Engine Performance Characteristics of Nanoparticles in Biodiesel-Based Fuels

##### Brake Thermal Efficiency

By comparing neat biodiesel to Ag-ZnO/ZnO-biodiesel, Sam Sukumar et al. [146] showed that the BTE of the nano-based fuels increased by 24% and 19.35%, respectively. According to the authors, this observation was as a result of the higher surface area and reactive surfaces of the nanoparticles, which generated maximum chemical reactivity within the fuel. Karthikeyan et al. [99] investigated the effect of cerium oxide additive on performance and emission characteristics of a CI engine operating on rice bran biodiesel and its blends. It was revealed that the presence of CeO_2_ nanoparticles enhanced proper fuel mixing and reduced fuel consumption, which consequently led to an increase in BTE compared to the neat base fuels. Baluchamy and Karuppusamy [103] investigated the combined effect of cobalt chromite nanoparticles and variable injection timing of preheated biodiesel and diesel on performance, combustion, and emission characteristics of CI engine. Their study showed that by advancing ignition timing, the BTE of blends SIT KC1 -ADV, SIT KC2 -ADV, SIT KC3 -ADV, and SIT KC4 -ADV increased by 3.2%, 3.7%, 4.5%, and 7.2%, respectively, when compared with the 23 CAD bTDC (i.e., standard injection timing) due to the presence of nanoparticles in the fuel and fuel burns completely during the combustion process. Various nano-additives (cerium oxide, zirconium oxide, and titanium oxide were blended in Garcinia gummi-gutta biodiesel–diesel blends (B20) for a comparative study conducted by Janakiraman et al. [97]. It was seen that adding metal oxide-based nano additive to B20 fuel reduced ignition delay and showed a slight rise in the net heat release rate and cylinder pressure, causing the BTE of the nano-based blends to increase compared to neat B20 fuel. Kumaran et al. [147] showed that 100 ppm of methanol-based hydroxyapatite nanorods has the ability to increase the BTE of waste cooking oil biodiesel as a result of the improved combustion atomization and rapid evaporation associated with the nanoparticles. 

##### Brake Specific Fuel Consumption

According to Debbarma and Misra [148], the presence of iron nanoparticles in biodiesel–diesel blends reduces energy consumption at full load engine conditions compared to the other test fuels without the nanoparticles. They explained that the presence of the iron nanoparticles increased the calorific value of the base fuel to generate some intensity of power with low consumption of fuel. However, increasing the concentration of the iron additives in the modified biodiesel will increase the energy consumption due to the increase in density and viscosity of the fuel in the presence of a higher concentration of nanoparticles. Nano-copper additive was added to *Calophyllum inophyllum* by Tamilvanan et al. [149] to investigate its effect on the performance, combustion, and emission characteristics of a CI engine. According to the researchers, a reduction of 3–6% in BSFC was achieved for biodiesel blends with the copper additives compared to a neat biodiesel blend at maximum load. They explained that the presence of the metal additive may have caused an increase in combustion temperature, which led to an increase in the conversion efficiency of heat energy into mechanical work and resulted in a reduction in BSFC. Rice husk nanoparticles (0.1%) were blended into B10 and B20 neem oil biodiesel-diesel blends by Sivasaravanan et al. [150]. The addition of rice husk nanoparticles slightly reduced BSFC when added to B10 and B20, in the range of 3.8–6.9% and 2.5–6.1%, respectively. The presence of the rice husk nanoparticle improved the combustion efficiency of all test fuels, and hence lower fuel was required to produce the same amount of work as that of the test fuels without nanoparticles. In a comparative study of two different carbon-based nano-additives, Chacko and Jeyaseelan [105] used graphene oxide and graphene nanoplatelets as fuel additives in diesel and biodiesel blends. Results revealed that by adding the nano-additives, the BSFC of the base fuels was positively impacted. According to the authors, the nanoparticles reduced ignition delay and combustion duration to ensure efficient combustion of the fuel supplied. It was also reported that the graphene oxide-based blends had a better BSFC than the graphene nanoplatelets-based blends. The former blend possessed a higher in-cylinder pressure which in turn produces better power output than the combustion with the latter blends. Microalgae biodiesel produced from Botryococcus braunii algal oil were doped with a mixture of titanium dioxide and silicon dioxide nanoparticles at dosages of 50 and 100 ppm in the work of Karthikeyan and Prathima [151]. Results from the study indicated that BSFC at all BMEPs were lower for B20 + 50 ppm (TiO_2_ + SiO_2_) and B20 + 100 ppm (TiO_2_ + SiO_2_) than diesel and B20 fuels. From the researchers’ point of view, the addition of the mixed nanoparticles oxidized the carbon deposits from the engine which led to an efficient operation and reduced fuel consumption.

##### Brake Power and Brake Torque

With the aim of investigating the effect of aqueous nanofluids on the performance and exhaust emissions of diesel engines, Khalife et al. [98] added cerium oxide into diesel-biodiesel–water blends (WBDE). Results revealed that adding cerium oxide nanoparticles in WBDE resulted in higher BP values compared with neat WBDE. The authors assigned this observation to the fact that metal-based additives could react with water at higher temperatures during the combustion process, resulting in hydrogen generation and, consequently, promoting the engine cylinder’s combustion process. Hoseini et al. [108] investigated the effect of graphene oxide nanoparticles on biodiesels from three different feedstocks; Evening primrose, Tree of heaven, and Camelina. It was seen in their results that the BP of all three biodiesels increases with the addition of the nanoparticles. Graphene oxide nanoparticles have the ability to increase the heat of evaporation of fuel. They have high energy content and a high surface-to-volume ratio. These properties lead to an increase in density of fuel–air charge, better oxidation of fuel blends, and high enthalpy of combustion, which caused an increase in the BP of the base fuels. It is also worth noting that, due to the high lower heating value of Camelina, its BP was greater than the other two test biodiesels. Alumina nanoparticles (40, 80, 120, and 160 ppm) were prepared and added as an additive to waste cooking oil biodiesel–diesel blend by Ghanbari et al. [152]. The process resulted in a significant increase in BT and BP. The researchers explained that this result was due to the improvement of the surface-to-volume ratio and catalytic effect of the alumina nanoparticles in the fuel blend, which improved combustion quality. In another study, palm–sesame biodiesel was blended with oxygenated alcohols in the presence of 100 ppm CNT and TiO_2_ nanoparticles [153]. Compared to B30 fuel, B30+ TiO_2_ and B30 + CNT blended fuels showed a slight decrease in average BT values by 1.28% and 0.88%, respectively. This was a result of an increase in viscosity and density values of the base fuel with the addition of the nanoparticles. The trend was quite similar to BP. Compared to B30 fuel, B30 + TiO_2_ and B30 + CNT blended fuels showed a slight decrease in average BP values by 1.47% and 1.04%, respectively. According to the study of Shekofteh et al. [154], functionalized MWCNTs–OH were blended into diesel–biodiesel–bioethanol blends for performance and emission characteristics. MWCNTs–OH into the base fuel improved BT and BP. In comparison to D100 and B5, adding MWCNTs–OH to B5E4 and B5E8 at 1800 rpm resulted in an increase in torque of 8.61 and 7.41 percent on average. Similarly, when MWCNTs–OH was added to B5E4 and B5E8 fuels at 2400 rpm, the torque increased by 14.19 and 11.32 percent, respectively, as compared to D100 and B5. As MWCNTs–OH was added to B5E4 and B5E8 at 1800 rpm, power increased by 7.33 and 4.35 percent, respectively, when compared to D100 and B5. Similarly, adding MWCNTs–OH to B5E4 and B5E8 at 2400 rpm increased power by 18.90 and 17.71 percent, respectively. The observed findings, according to the researchers, were attributable to the inclusion of the nanoparticles, which generated greater peak cylinder pressure and a faster heat release rate by lowering the ignition delay and combustion duration of fuel in the engine, resulting in a more complete combustion of the engine.

#### 4.2.8. Engine Emission Characteristics of Nanoparticles in Biodiesel–Based Fuels

##### Carbon Monoxide

In the study by Anbarasu and Karthikeyan [101], it is seen that cerium oxide blended biodiesel emulsion fuels recorded a significant reduction in CO emissions compared to the other test fuels. The presence of the cerium oxide in the base fuel shortened ignition delay and improved combustion characteristics. As a result, there was an improvement in the quantity of fuel–air mixing and uniform burning. Srinivasan et al. [155] studied the effect of Al_2_O_3_ and TiO_2_ of dosages 25 ppm and 50 ppm on a diesel engine fueled with rubber seed oil methyl ester. The addition of the nanoparticles resulted in a reduction in CO from 0.31% to 0.76% and 0.3% to 0.75% by adding 25 ppm and 50 ppm of Al_2_O_3_ to B100, respectively. On the other hand, adding 25 ppm and 50 ppm of TiO_2_ to B100 led to a reduction in CO emissions from 0.29% to 0.74% and 0.28% to 0.73%, respectively. According to the researchers, the nanoparticles presented additional oxygen molecules to facilitate oxidation of reaction, leading to better combustion and decreased CO emissions. Additionally, the nanoparticles underwent a catalytic oxidation reaction, which improved the mixing rate of air with fuel. Nithya et al. [96] made an investigation into the effect of engine emission operating on canola biodiesel blends with TiO_2_. Their results revealed that by adding the nanoparticles to B20 fuel, the CO emission decreases nearly 30% at full engine load due to the shorter ignition delay and provision of additional oxygen molecules by the nanoparticles, which helped achieve a more complete combustion. Orange peel oil biodiesel was converted to a nanofluid under the influence of titanium dioxide by Kumar et al. [106] for assessing its effects on the performance, emission, and combustion characteristics of a diesel engine. It was reported in this work that at peak power output, the CO emissions for diesel fuel, pure OOME, OOME–T50, and OOME–T100 were 0.58%, 0.55%, 0.51%, and 0.45%, respectively. The authors claimed that the ability of titanium dioxide to act as an oxidation catalyst offered more oxygen for the burning of the fuel inside the chamber, which resulted in complete combustion and reduced the creation of CO emissions. Cerium oxide and Gadolinium doped cerium oxide (CeO_2_:Gd) nanoparticles were dispersed in blended Pongamia oil biodiesel by Dhanasekar et al. [156] to analyse the emission from a four stroke single cylinder diesel engine. Results showed that compared to pure biodiesel, the CO emission is drastically decreased in the ceria and GDC blended fuels as a result of the nanoparticles’ reaction with surface oxygen which is released from cerium oxide and GDC nanoparticles. The investigators also reported that, GDC nanoparticles showed better CO emission reduction pure diesel, which may be due to the high content of surface oxygen of GDC nanoparticles as it converts CO into CO_2_.

##### Hydrocarbons

Copper (II) oxide nanoparticles were added to Jojoba biodiesel blend (JB20) by Rastogi et al. [94] to investigate its effect on the performance and emission characteristics of a diesel engine. It was reported from this work that the CuO had a significant impact on reducing HC emissions of the base fuel. The average HC emissions for the JB20, JB20CN25, JB20CN50, and JB20CN75 were reduced by 5.18%, 9.39%, 12.17%, 7.45% with respect to diesel fuel at 5.2 kW engine load. The results were ascribed to the excellent characteristics introduced by CuO such as, increase in calorific value, decreasing fuel viscosity due to which proper fuel atomization occurred, better fuel explosion process, shortened ignition delay, and enhanced heat release rate during fuel combustion, which promoted complete combustion process inside the combustion chamber. Ramakrishnan et al. [102] presented findings to show the role of nano additive blended biodiesel on emission characteristics of a diesel engine. The authors added carbon nanotubes to neem biodiesel (NBD) and observed that the addition of CNT at 50 ppm and 100 ppm to NBD reduced HC emissions by 5.1% and 6.7%, respectively, at all loads. The authors gave the following reasons for the obtained results for HC emissions: (1) the positive effect of CNT, which acted as an oxidation catalyst, lowers the carbon combustion activation temperature and improve the oxidation of NBD, and (2) CNT reduces ignition delay and improves secondary atomization leading to enhanced combustion. Solmaz et al. [104] predicted the performance and exhaust emission characteristics of a CI engine fuelled with MWCNTs doped biodiesel–diesel blends using response surface methodology. They reported that exhaust HC concentrations of the test fuels decreased with the addition of the MWCNTs into B20 fuel. The authors argued that the improved combustion characteristics and catalyst activity of MWCNTs were responsible for such decrement in the exhaust HC concentrations. In the experiment of Gad and Jayaraj [157], carbon nanotubes, titanium dioxide, and aluminum dioxide of concentrations 25, 50, and 100 ppm were blended into Jatropha biodiesel–diesel blend. The maximum decrease in HC emissions of J20C25, J20C50, J20C100, J20T25, J20T50, JT20T100, J20A25, J20A50, and JT20A100 were 4%, 12%, 15%, 22%, 17%, 15%, 19%, 21%, 18%, respectively, compared to neat diesel. The decrease in HC emissions for the nanofuels was attributed to the higher catalytic activity and the higher surface-to-volume ratio of the nanoparticles as well as their ability to lower carbon combustion activation temperature and enhance oxidation of fuel. Further, 25 and 50 ppm of MWCNT nanoparticles were blended in Honge oil methyl ester (HOME) by Tewari et al. [109]. HC emission for HOME operation was higher compared to diesel but lower for the HOME–MWCNTs than pure HOME. The HC emissions for HOME50MWCNT, HOME25MWCNT, HOME and for diesel were 58, 70, 82, and 32 ppm at 80% load, respectively. The lower HC emissions for the MWCNT–blends were lower than pure HOME due to the catalytic activity and improved combustion characteristics of MWCNT which promoted complete combustion.

##### Nitrogen Oxides

El-Seesy et al. [92] revealed that the addition of MWCNTs to JB20D leads to a decrease in NO_x_ emissions compared to neat JB20D. The researchers attributed this trend to the catalytic effect of the MWCNTs that may have accelerated the combustion process to be completed, forming final products with a minimum thermal breakdown of the hydrocarbon compounds. To analyse the emission and performance of a direct injection engine fuelled with Mahua biodiesel blends, Rastogi et al. [95] blended Al_2_O_3_ nanoparticles into the base fuel. They showed that with the addition of the nanoparticles, the NO_x_ emissions of the blended fuels were lower than neat diesel. They accounted for this observation by explaining that the role Al_2_O_3_ plays in increasing surface area, reducing ignition delay, helping the active reaction of hydrocarbon with oxygen, and reducing the reaction of nitrogen with oxygen was the cause for the reduction in NO_x_ formation in the cylinder. The effect of 50–150 ppm of MWCNTs in the diesel–biodiesel blend was studied by Alenezi et al. [93] with a focus on the emission and combustion characteristics of diesel engines. It was shown in this work that the addition of MWCNTs to B20 and B40 base fuel resulted in a significant reduction in NO_x_ formation. However, the opposite trend of NO_x_ emissions was reported for B10 when the concentration of the MWCNTs was increased. Sulochana and Bhatti [107] added MWCNTs in 25 ppm and 50 ppm mass fractions to waste fry oil biodiesel. It was highlighted that, due to their higher premixed combustion heat release rate and complete combustion, WFOME25MWCNT produced higher NO_x_ emissions than WFOME50MWCNT and pure WFOME. The recorded emission of NO_x_ for diesel, WFOME, WFOME50MWCNT and WFOME25MWCNT were 654 ppm, 731 ppm, 764 ppm, and 884 ppm, respectively. In the work of Deepak Kumar et al. [158], biodiesel derived from cottonseed oil has been investigated along with 80 ppm of ZnO. NO_x_ emission of ZnO-based fuels was lower compared to pure diesel and biodiesel. In their work, the authors explained that zinc oxide raises the average temperature of the combustion chamber (due to its higher calorific value), allowing more oxygen in the mixture to react, resulting in fewer NO_x_ emissions. For NO_x_ reduction, zinc oxide absorbs oxygen. Shorter ignition delays result in better fuel–air mixing, which results in an oxygen deficit for NO_x_, lowering NO_x_ emissions. 

#### 4.2.9. Effect of Nano-Additives and Diesel–Biodiesel Blends on Engine Combustion

This subsection critically evaluates the combustion effect of diesel fuel blended with different biodiesel and nano-additives in various CI engines. The most recent studies on diesel/biodiesel–nanoparticle blends discovered that the combustion of such mixture in diesel engines could offer many remarkable outputs such as better atomization behavior [129], good solubility, and a higher surface to volume ratio [104,106], low combustion activation temperature due to high thermal conductivity and catalytic activity [126], shorter ignition delay [157], and high burning rate due to the improved latent heat of vaporization [159].

The key factors in evaluating the combustion characteristics are in-cylinder pressure rise rate and heat release rate [160]. As recently reported, the ignition delay increases with an increase in diesel–biodiesel blend fuels [97,161]; this was accompanied by a large amount of fuels in the premixed combustion phasing [27], thereby resulting in minimal thermal efficiency [162]. This was due to the improvement in cetane number, viscosity and density of the blend fuel. This trend is in conformity with the result found with Al_2_O_3_, CeO_2_, Co(Al, Cr)_2_O_4_, and SiO_2_ [103,163,164,165], which reported fast burning rate during premixed combustion phase, resulting in high peak cylinder pressure with an improved heat release rate, as summarized in Table 6. This occurrence is inconsistent to the final remark found by Ranjan et al. [166] with the addition of MgO NPs and [167] with ZnO NPs, which had a low ignition delay due to lower viscosity. The result was later accompanied by a low heat release rate and a decrease in peak cylinder pressure.

Improving the conversion efficiency of the engine using nano-catalyst is a novel concept that was found suitable for a modified and unmodified engine. Janakiraman et al. [97] evaluates the effect of ZrO_2_ at 25 ppm concentration into *Garcinia* biodiesel (B20) to enhance the diesel engine efficiency. It was found that adding ZrO_2_ NPs, enhanced the ratio of surface area to volume, hence improving the HRR and ignition properties of the fuel blend. A similar trend in HRR was observed using biodiesel-based fuels on diesel engines with the addition of Al_2_O_3_ [168,169], CeO_2_ [159], and TiO_2_ [106]. Several researchers, such as [27,101,161,170,171] investigated and reported that the rationale behind the trend of HRR is similar to that of ICR in most cases.

## 5. Comparative Strengths of Different Nanoparticles in Same Base Fuel

Although nanoparticles can significantly influence the behaviour of fuels during combustion, the degree of improvement varies from one nanoparticle to another. With respect to their own unique characteristics, some nanoparticles perform better than their counterparts upon their addition in the same base fuels. For example, Ağbulut et al. [70] have shown that when Al_2_O_3_ and TiO_2_ of 100 ppm are blended in diesel–ethanol fuel, Al_2_O_3_ exhibits better performance and combustion characteristics than TiO_2_. This conclusion has also been reached in the study of Purushothaman et al. [125] where Al_2_O_3_ and TiO_2_ of 100 ppm were blended in Mahua oil. CNT, Al_2_O_3_ and TiO_2_ (50 ppm) were used as fuel additives in biodiesel–diethyl ether blends by Mardi K et al. [115]. At 1700 rpm, CNT showed better performance and emission characteristics than the other two nanoparticles, except for NO_x_ emissions, which were positively impacted the most by Al_2_O_3._ However, an opposite result to Mardi K et al. [115] has been reported by Gad and Jayaraj [157]; using the same nanoparticles in Jatropha biodiesel–diesel blends, at maximum load, Al_2_O_3_ showed the best characteristics for BSFC and BTE compared to CNT and TiO_2_, whereas CNT was best suited for CO and NO_x_ emissions. For HC emissions, TiO_2_ was the best nanoparticle. By doping ZnO with silver (Ag) nanoparticles for Pongamia biodiesel–diesel fuel, Sam Sukumar et al. [146] showed that Ag-ZnO had a better effect on engine characteristics than neat ZnO. Finally, Chacko and Jeyaseelan [105] used graphene oxide and graphene nanoplatelets as blend components in Karanja oil biodiesel/waste cooking oil biodiesel–diesel blends. It was reported in this study that, at 2250 rpm and BMEP of 3.45 bar, graphene oxide showed a better effect on engine performance, whereas graphene nanoplatelets were more favourable for engine emission reduction.

In general, for one nanoparticle to perform better than another, it means that the superior nanoparticle has enhanced catalytic activity, higher surface to volume ratio, better oxygen buffering, higher evaporation rate, and higher thermal conductivity compared to the inferior nanoparticles. Other factors such as nanoparticle size, viscosity, and density could also vary the performance of nanoparticles. However, it is worth noting that, by varying the concentrations of the base fuels and nanoparticles or engine operating conditions, a superior nanoparticle could become relatively inferior, and vice versa. 

## 6. Similarities and Differences in Engine Characteristics of the Same Nanoparticle in Low-Carbon Fuels 

During our examination, we noticed several comparable and contrasting themes across the diverse research studies. This section describes the similarities and differences in engine characteristics for all three liquid fuels when blended with the same nanoparticle under three independent and varying experimental conditions, including engine load and speed, nanoparticle concentration and size, and base fuel concentration. The addition of iron oxide nanoparticles in alcohol [69], vegetable oil [91], and biodiesel [172] were all reported to have led to a decrease in CO, NO_x_, and HC, while BTE was increased in all three cases. According to Ağbulut et al. [70], Chinnasamy et al. [88], and Anchupogu et al. [111], the addition of alumina nanoparticles to alcohol, bio-oil, and biodiesel, respectively, results in a decrease in HC and CO. However, NO_x_ emissions increase in the bio-oil while they decrease in the alcohol and biodiesel. Cerium oxide in alcohol [75], vegetable oil [85], and biodiesel [97] resulted in a CO, HC, NO_x_, and increase in BTE. However, unlike alcohol and biodiesel, BSFC worsened in vegetable oil. Örs et al. [81], Elumalai et al. [129], Kumar et al. [106] to alcohol, vegetable oil, and biodiesel, respectively, BSFC, CO, HC reduced, but NO_x_ increased in the alcohol and vegetable oil systems whilst it reduced in the biodiesel fuels. It was shown in the experimental findings of Wei et al. [74] (alcohol) and Gavhane et al. [173] (biodiesel) that upon the addition of silicon dioxide nanoparticles, CO, HC, smoke reduces and BTE increases. However, the opposing trend was that, in the alcohol system, BSFC and NO_x_ improve while they worsen in the biodiesel system. By blending MWCNT in alcohol [68], bio-oil [141], and biodiesel [107], NO_x_ emissions were reported to have increased in all three experiments. Results from El-Seesy and Hassan [77] and Chacko and Jeyaseelan [105] revealed that when GO/GNP nanoparticles are blended in alcohol and biodiesel-based fuels, respectively, NO, CO, and HC emissions of the base fuels reduce significantly. In the works of Heydari-Maleney et al. [65] (alcohol), Sharma et al. [90] (bio-oil), and Ramakrishnan et al. [102] (biodiesel), all three investigations reported that CO and HC decrease upon the addition of CNT. However, unlike the bio-oil and biodiesel, NOx of the alcohol fuel increased. The analysis made in this section also reveals that, though nanoparticles can positively or negatively impact the performance, emission, and combustion characteristics of the base fuels, the type or extent of impact depends on certain inherent factors such as the type of nanoparticle, type of base fuel, concentration and size of nanoparticle, engine conditions such as load and speed, and the approach in which the nanoparticles were prepared and blended into the base fuels.

## 7. Summary of the Mechanism Involved with Nanoparticle’s Role during Low Carbon Fuel Combustion in ICE 

Evidence from literary sources, as presented in the previous sections, points to one obvious fact: nanoparticles generally produce better engine performance, combustion, and emission characteristics when blended in liquid biofuels. However, as mentioned earlier, the extent of improvement will significantly be determined by the type of nanoparticle, type of base fuel, concentration and size of the nanoparticle, engine conditions such as load and speed, and the approach in which the nanoparticles were prepared and blended into the base fuels. Various researchers have given several reasons to explain how these oxides of metal and carbon nano-additives improve the engine characteristics upon their addition into the base fuels. In this section, we only present more general and the most consistent reasons given by investigators of the reviewed literature in the previous sections. First of all, nanoparticles play a role as oxygen buffers. By doing so, additional oxygen molecules are provided in the combustion chamber, which promotes complete combustion and lowers unburnt emissions. Secondly, most of the reviewed nanoparticles have a higher surface area to volume ratio. This enhances catalytic behaviour by providing a larger surface area for the fuel particles to interact and also produce more energy inside the cylinder, which provides an efficient burning process to obtain a more complete combustion and reduced emission of pollutants. Next the nanoparticles exhibit micro-explosive properties, thereby promoting better atomization and air–fuel mixing. Furthermore, due to their high thermal conductivity, nanoparticles can act as heat sinks which helps decrease the temperature and NO_x_ emissions. In addition, nanoparticles show catalytic activity, which lowers combustion activation temperature and helps increase the burning rate. Moreover, there is a higher evaporation rate with nanoparticles which leads to an enhanced mixture of fuel vapour with air, reducing ignition delay and combustion duration to increase the chances of complete combustion. Nanoparticles can also act as an oxidation catalyst that promotes the oxidation of hydrocarbons to reduce HC emissions.

Some authors have also reported other factors such as surface tension and latent heat of vaporization of nanoparticles to be associated with the effective combustion of nanofluids. The wettability of the fluid improves as the surface tension is reduced. Spray parameters such as droplet size, dispersion, and spray angle are heavily influenced by surface tension in combustion applications. One of the most important factors in influencing the burning rate of liquid fuels is the latent heat of vaporization. This is an important result since a greater burning rate suggests more efficient combustion and maybe a smaller combustor, and this can be dramatically altered when the latent heat of vaporization of nanofluids is varied.

Depending on several conditions such as fuel concentration, dosage and size of nanoparticles, experimental setup, and researcher(s)’ experience, each nanoparticle affects the physicochemical properties of base fuels differently. In other words, for the same nanoparticle, fuel property adjustment varies in trend from one study to another. However, a general trend could still be observed in the reported studies. For metals and their oxides, titanium and aluminum have proven to be excellent additives for enhancing the calorific value and cetane number of the base fuels. Similarly, amongst the carbon-based nanomaterials, it would appear that the multi-walled carbon nanotubes are more suited for improving the energy content and ignition qualities of the base fuels. For viscosity and density, the general trend shows slight increase in the values of the base fuels after the addition of the nanoparticles. It is advisable for researchers to carefully consider the type of base fuel and its concentration, the dosage and size of the nano-additives, and the specific targets of the study before selecting a particular nanoparticle(s) for modifying or designing any new fuel. This would ensure a more consistent and reliable trend in the fuel properties adjustment of nanofuels. 

## 8. Exergy, Exergoeconomic, Exergoenvironmental, and Sustainability of Nano-Additives and Low Carbon Fuels in ICE

**Figure 7:**
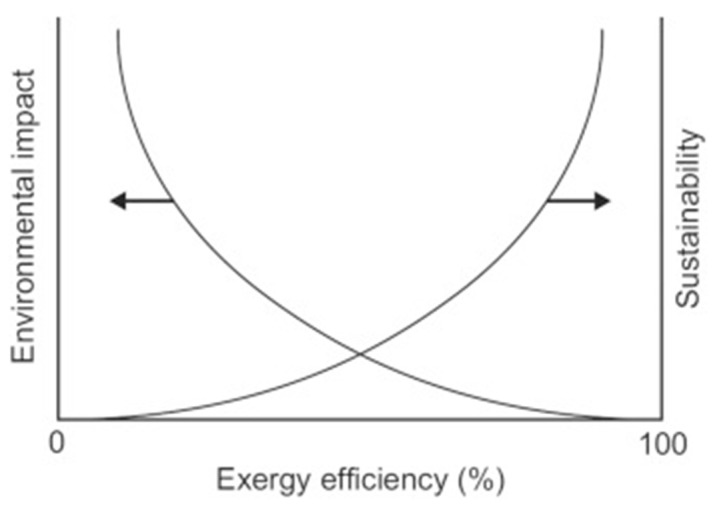
Intercourse between exergy efficiency, environmental impact, and sustainability of a thermal system. (Reprinted with permission from Ref. [177] with permission. Copyright © 2001 Elsevier.

In the previous sections, the energy-based analysis such as BTE, BSFC, BT, and BP as well as the emission characteristics of diesel engines fuelled with low carbon fuels under the influence of nano-additives have been discussed. Indeed, these energy-based indicators have been extensively studied and reported in literature. Energy analysis has been criticized in the open literature for failing to consider the effect of second law’s limitation on an energy conversion process [36]. On the other hand, exergy analysis fills this gap by providing relevant information on the irreversibility aspects (availability losses) of energy conversion systems [176]. Evaluating the performance of thermal systems based solely on exergy analysis is not sufficient, and the analysis could be more comprehensive when economic, environmental, and sustainability aspects are included. These aspects put together provide a complete understanding on the profitability and sustainability of an improvement achieved through exergy analysis. [35]. Figure 7 shows the nexus between exergy efficiency, environmental impact, and sustainability of a thermal system [177].

From Figure 7, it is observed how, by increasing exergy efficiency of a process, there is a corresponding improvement in system sustainability and reduction in environmental impact [177]. As environmental impact approaches zero, exergy efficiency approaches 100% and, simultaneously, sustainability is promoted by virtue of the fact that the process approaches an ideal reversible process [36]. Furthermore, reduction in exergy efficiency towards 0% affects the sustainability negatively (also approaches zero). This is because the exergy-containing resources are being utilized but there is no meaningful outcome. In this same direction, environmental impact approaches infinity since for a provision of fixed service, an ever-increasing amount of resources must be consumed which leads to the creation of exergy-containing waste by the same magnitude [177]. In order to prevent wrong decisions while evaluating modification of thermal processes, it is important to consider the multidirectional analysis of the thermal systems. This will create an environment for accurately determining the quantity and quality of nanofuels used in ICE.

In the last five years, a limited number of studies have been conducted on nanofuels in diesel engines based on several versions of exergy analysis. Table 7 summarizes the main findings from these studies.

According to the reviewed literature, in general, per the second law of efficiency, results have shown that the thermal system performance and sustainability of low carbon fuels blended in diesel becomes worse compared to pure diesel. This could be attributed to relatively inferior properties of the low carbon fuels such as higher viscosity, cetane number, latent of vaporization, and lower calorific value, resulting in the engines’ poor combustion characteristics. Nonetheless, low carbon fuels such as alcohols and biodiesels help reduce overdependence on fossil fuels for transport applications. Hence, their use is still ongoing and a major research hotspot. The existing studies have shown that the aforementioned situation significantly improves when the base fuel is modified with nanoparticles. The surface area, catalytic activity, and oxygen buffering of these NPs are very favourable for improving the ignition qualities of the base fuel, accelerating chemical reactions, promoting complete combustion, and enhancing the thermal properties. These events work together to enhance exergy efficiencies, reduce unaccounted thermal losses and entropy generation. Against this backdrop, other exergy indicators such as exergoeconomic, exergoenvironmental, and sustainability of the nanofuels become more optimal compared to the base fuels. Nanoparticle size and dosage also affect the performance and sustainability of thermal systems fuelled with nanofuels. The main challenge now has to do with the production of nanoparticles which is quite an expensive venture in today’s market. However, as research continues to improve, the unit price of nano-additives could be greatly reduced in the near foreseeable future, further bolstering the feasibility and attractiveness of nanofuels from a technical, economical, and environmental point of view.

## 9. Toxicity and Health Impacts of Nanoparticles

**Figure 8:**
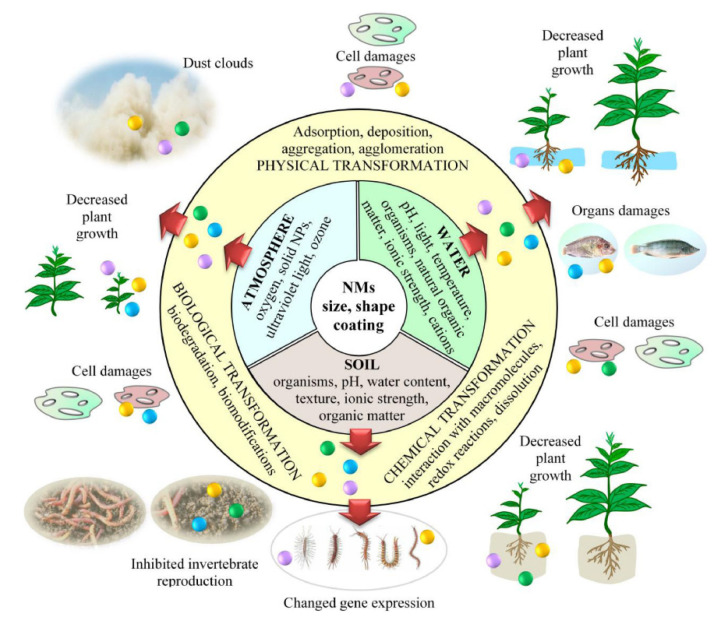
Toxicity and health impact of NPs. (Adapted with permission from Ref. [25]. Copyright © 2022 Elsevier.)

One of the primary obstacles to broad commercial deployment of these nano-additives, as with many nanomaterials, is their potential toxicity and health effects. Nanotoxicology is concerned with the research and improved understanding of nanoparticle toxicity. Nanoparticles are significantly linked to toxicity, according to several in vivo and in vitro studies. Despite the advantages of nanoparticles, humans, animals, and plants have been exposed to their potential toxicity through different nanotechnology applications (see Figure 8).

Inhalation, ingestion, skin absorption, injection, and implantation are among ways that nanoparticles can enter the human body [179]. Due to the small size of nanoparticles, their ease of penetration and biocompatibility, and their potential ability to breach the placental barrier, the widespread use of nanomaterials has raised concerns about their negative impact on human health, particularly on men’s and women’s reproductive systems, as well as fetal health. Early research on anthropogenic nanoparticles, such as diesel exhaust, shows that they aggregate and attach to human cells as a result of regular exposure, causing disruption to normal physiological processes. Moreover, nanoparticles have been linked to pulmonary injury, hepatotoxicity, immuno-nanotoxicity neurotoxicity, renal toxicity, and permanent testicular damage in animals [180]. Nanomaterials can clump together to form larger particles or longer fibre chains, altering their characteristics and potentially affecting their behaviour in both indoor and outdoor environments, as well as their potential exposure and entrance into the human body [181]. Due to large surface area, high surface activity, unique shape, tiny diameters, or decomposition into smaller particles after deposition, they might deposit in the respiratory system and exhibit nanostructure-influenced toxicity. If nanomaterial-derived particles display nanostructure-dependent biological activity, they may pose a danger. Nanoparticles have high deposition efficiency in healthy people’s lungs, and much greater deposition efficiencies in those with asthma or chronic obstructive pulmonary disease [182,183]. When nanoparticles are breathed, they deposit dispersedly on the alveolar surface, causing a scattered chemo-attractant signal and lowering identification and alveolar macrophage responses. Karlsson et al. [44] looked into the cytotoxicity and capacity to produce DNA damage and oxidative stress of various nanoparticles and nanotubes. Their research evaluated the toxicity of metal oxide nanoparticles (CuO, TiO_2_, ZnO, CuZnFe_2_O_4_, Fe_3_O_4_, Fe_2_O_3_) to carbon nanoparticles and multi-walled carbon nanotubes (MWCNT). Cell viability and DNA damage were both affected by ZnO, but DNA damage was solely induced by TiO_2_ particles (a combination of rutile and anatase). No or low toxicity was reported for iron oxide particles (Fe_3_O_4_, Fe_2_O_3_), while CuZnFe_2_O_4_ particles were rather effective in causing DNA damages. Finally, even at the lowest dose tested, carbon nanotubes were cytotoxic and caused DNA damage.

External nanoparticles comprising zinc and aluminum have recently been shown to have harmful effects on seedling germination and root growth in a range of plant species according to Lin and Xing [45] and Doshi et al. [43]. They found that when ZnO nanoparticles were exposed to the root surface, Ryegrass biomass was reduced substantially, root tips shrunk, and root epidermal and cortical cells were severely vacuolated or collapsed. Further, according to Soutter [48], diesel fuels enriched with cerium oxide nanoparticles have been observed to produce pulmonary consequences in exposed rats, including increased bronchial alveolar lavage fluid and lung inflammation. Long et al. [46] reported that titanium dioxide nanoparticles found in sunscreens could cause brain damage in mice. Nano size titanium dioxide stimulates reactive oxygen species in brain microglia and damages neurons in vitro [47]. Balasubramanyam et al. [184] reported that aluminum oxide nanoparticles (30–40 nm) contain genotoxic characteristics that are dosage-dependent. They used rat blood cells to test for genotoxicity using the comet assay and the micronucleus test. Another study employing a mouse lymphoma cell line found that aluminum oxide nanoparticles (50 nm) have genotoxic effects in the form of DNA damage without being mutagenic [185]. Titanium dioxide possesses some toxic health effects in experimental animals, including DNA damage as well as genotoxicity and lung inflammation [186,187]. Titanium dioxide nanoparticles (<100 nm) induce oxidative stress and form DNA adducts [188]. Titanium dioxide nanoparticles (5–200 nm) are harmful to immune function, liver, kidney, spleen, myocardium, hyperglycaemia, and lipid homeostasis in experimental animals, in addition to genotoxicity [189,190]. In vivo investigations have demonstrated that iron oxide nanoparticles stay in cell organelles (endosomes/lysosomes) after entering the cells, decompose in the cytoplasm, and contribute to cellular iron pool [191]. After inhalation, magnetic iron oxide nanoparticles were shown to collect in the liver, spleen, lungs, and brain. Murine macrophage cells, human macrophages, human hepatocellular carcinoma cells, and rat mesenchymal stem cells were all found to be at risk after exposure to the nanoparticles made from iron oxide. On murine macrophage cells, iron oxide nanoparticles were found to be lethal at concentrations of 25–200 g/mL after a 2-h exposure. Their study also reported consequences such as a reduction in cell viability [192]. On human brain microvascular endothelial cells (HBMVECs), aluminum nanoparticles in the size range of 1–10 µM were utilized for 24 h. Treatment led in a decrease in mitochondrial activity, cell viability, and an increase in oxidative stress, according to the research of Chen et al. [193]. The effect of MWCNTs was also evaluated in a study conducted on rat [194]. Rats were exposed to MWCNTs intratracheally in this study. Both histologically and biochemically, the researchers looked at inflammation, lung persistence, and fibrotic responses. The bronchial lumen was found to have pulmonary lesions, which were characterized by collagen-rich granulomas. Cha and Myung [195] tested the cytotoxicity of zinc, iron, and silicon at various doses against cell lines from the liver (Huh7), brain (A-172), stomach (MKN-1), lung (A-549), and kidney (HEK293). The decrease in DNA content, as well as mitochondrial activity, was easily detected in brain and liver cells. In a research of zebrafish embryos (Danio rerio), Asharani et al. [196] discovered that uncoated silver nanoparticles caused higher genotoxicity because they were able to reach the nucleus cells, causing DNA strands to break.

It is evident from the reviewed works that almost all nanoparticles are closely associated with toxicity and have shown to have detrimental health impacts. Over-exposure to nanoparticles has been proved to cause DNA and reproductive damage, cytotoxicity, and even cancer. There are currently ongoing studies aimed at providing better nanotoxicity evaluation and measures to minimize nanotoxicity levels in the environment. Other areas of research are still being conducted to mitigate the dangers posed by metal nanoparticles in the production of biodegradable and biocompatible nanoparticles. The current state-of-the art is thus on the development of nanoparticles that interact better with the environment and have less harmful effects.

It is worth mentioning that studies have also made conscious attempts to reduce the toxicity of nanoparticles. Some of the approaches include degradable nanoparticles, next generation lipids, surface coating, doping, and alteration of surface properties [197]. Doping of nanoparticles with dopants such as aluminum titanium and iron has been found to decrease nanoparticle dissolution and cause a reduction in toxic ions released, and this would cause an alteration to the reactive surfaces leading to a decrease in reactive oxygen species generation [197,198]. On the other hand, surface coating is an approach for modifying or diminishing the adverse effects associated with nanomaterials. It includes modifying properties such as stability of nanoparticles, agglomeration and arrest dissolution and discharge of noxious ions [199]. Cai et al. [200] have reported that ethylenediamine tetra coating could passivate the surface of metal oxides, thereby reducing their toxicity and pulmonary hazard effect. Methods focusing on altering properties of nanoparticles to reduce their toxicity also include alteration of surface charge, aggregation characteristics and/or hydrodynamic diameter of nanoparticles [201]. There are, however, ongoing studies to improve the efficacy of the abovementioned methods.

## 10. Conclusions and Future Research Direction

As automotive industries continue to look for more efficient combustion of liquid fuels coupled with the existence of stringent environmental regulations, nanoparticles as fuel additives for combustion in diesel engines have become an important research field in recent years. Several studies have been conducted to review the effect of nanoparticles on the performance, emission, and combustion characteristics of liquid fuels. However, these studies, to a large extent, have primarily focused on biodiesel, whereas those on vegetable oils and alcohols remain scarce. In our quest to bridge the existing gap in the literature, the current study was set out to simultaneously and holistically review experimental results related to all three biofuels (alcohols, biodiesels, vegetable oils) in the context of the effect nanoparticles may have on their fuel properties, performance, emission, and combustion characteristics when operating in a diesel engine. Another novelty presented in this work relates to the evolutionary trends, research hotspots, and key contributors of this research field from 2000 to 2021. Of the three biofuels reviewed, biodiesels have been the most investigated on how they perform in diesel engines under the influence of nanoparticles. Earlier research focused extensively on carbon nanotubes, but the recent trend shows a shift towards cerium dioxide, titanium dioxide, and, mainly, aluminum dioxide. The key contributors to this field originate from Asia, largely represented by India, China, and Iran. It became apparent that the key interest of this research field hinges on the effect of nanoparticles on the performance, emission, and combustion characteristics of alcohol/biodiesel/vegetable oil-based fuels.

Nanoparticles can positively impact key physical properties of the base fuels such as density, kinematic viscosity, cetane number, flash point, calorific value, etc., but the extent of the impact will greatly depend on the type and size of the nanoparticle, type of base fuel, and concentration of blends. For performance characteristics, it is evident that most of the studies carried out with the addition of nano-particles into prospective renewable additives (such as biodiesel, vegetable-based oil, and alcohol) showed a significant reduction in BSFC, while BTE tended to increase. These were attributed to the improved fuel properties, excess oxygen content, better atomization, high thermal conductivity, and good catalytic activity of nanoparticles in renewable additives. For combustion characteristics, the heat release rate and in-cylinder pressure can either decrease or increase as investigated. These inconsistencies occur due to many factors: (i) when the ignition delay increases due to higher viscosity, the peak heat release rate and in-cylinder pressure increase, leading to high fuel droplets in the premixed combustion phase, and (ii) when the flame temperature inside the combustion increases due to low viscosity and ignition delay, the in-cylinder pressure, and thermal efficiency increase, promoting soot particles’ oxidation rate. However, most of the in-cylinder pressure and heat release rates significantly improved with nano-additives/renewable fuels in relation to control conditions. In most of the cases, the addition of nanoparticles showed a slight reduction in NOx due to an increase in the cooling effect of nanofluids with a substantial reduction in CO and HC emissions compared to diesel fuel. Various investigators attributed the role of nanoparticles in fuels to their oxygen buffering, the higher surface-to-volume ratio, micro-explosive property, thermal conductivity, evaporation rate, and oxidation catalysis. Based on these characteristics, there is efficient and more complete combustion of fuel to the positive impact the performance, emission, and combustion characteristics.

Beyond energy-based indicators, the exergy, economic, environmental, and sustainability aspects of the blends in diesel engines were discussed. It is observed that the performance of the diesel engine fuelled with low carbon fuels, according to the second law of efficiency, improves under the influence of the nano-additives. By virtue of their oxygen buffering, higher surface-to-volume ratio, micro-explosive property, thermal conductivity, evaporation rate, and oxidation catalysis, nanoparticles in low carbon fuels lead to high combustion efficiency and accelerated chemical reactions, which result in improved exergy efficiencies. In return, the exergoeconomic, exergoenvironmental, and sustainability aspects of these nanofuels are superior compared to the base fuels.

Some key recommendations and future perspectives are provided as follows. Contributions from some parts of Asia, South America, Africa, and Oceania are very underrepresented, and the most active researchers could attempt collaborative works with authors from these continents for more ground-breaking discoveries and development of this research field. Future studies should devote more attention to alcohols (especially > C2 alcohols) and vegetable oils. Researchers can also experiment on hybrid nanoparticles by blending multiple nanoparticles and studying how it affects engine characteristics. There is still more work that needs to be undertaken in the area of different nanoparticles in the same base fuel. The optimum concentration and size of the nanoparticles together with the base fuels for an efficient combustion and reduced emissions should be studied. Since certain nanoparticles are very surface reactive, long-term studies of the engine or engine exhaust resistivity are necessary. More studies are needed on the exergy, exergo-economic, exergo-environmental, and sustainability aspects of nanofuels in ICE to complement the highly existing energy-based studies. Furthermore, because some researchers have identified the cost of nanoparticles as an inherent problem, future research can also look at finding an optimum balance between the performance and cost of nanoparticles to increase their feasibility and wide use. Metal nanoparticles represent a threat to human health, and biodegradable and biocompatible nanoparticles may help to minimize this risk. As a result, the focus is on developing nanoparticles that interact better with the environment and have fewer negative consequences. It should be noted that nanoparticles are not directly released into the atmosphere. They are mixed as an additive in the fuel and go through a complex combustion process. The effect of the nanoparticle additives on the atmosphere after being combusted in the engine needs to be researched.

## Figures and Tables

**Table 1:** Summary of nanoparticles’ effect on fuel properties of alcohol-based fuels.

Alcohol	Case #	Fuel	Nanoparticle (DOSAGE)	Density (kgm^−3^)	Viscosity (mm^2^/s)	Flash Point (°C)	Calorific Value (MJ/kg)	Cetane Number
Ethanol [73]	1a	D40B30E30	Absent	828.5	2.42	10	39.90	57
	1b	D40B30E30	ZnO (250 ppm)	836.3	2.32	16	36.89	55
Methanol [74]	2a	M100	Absent	790	0.59	-	20.3	-
	2b	MSN25	SiO_2_ (25 ppm)	793	0.62	-	21.9	-
	2c	MSN50	SiO_2_ (50 ppm)	798	0.65	-	22.4	-
	2d	MSN100	SiO_2_ (100 ppm)	804	0.71	-	23.2	-
Pentanol [76]	3a	TF	Absent	841	3.3	3	41.62	48
	3b	TF40	SiO_2_ (40 ppm)	839	3.37	2.8	41.73	48.5
	3c	TF80	SiO_2_ (80 ppm)	837	3.21	4	41.96	55
	3d	TF120	SiO_2_ (120 ppm)	830	3.01	3	42.97	47.4
N-amyl [69]	4a	TF	Absent	841	3.3	3	41.62	48
	4b	TF40	Fe_2_O_3_ (40 ppm)	839	3.37	2.8	41.73	48.5
	4c	TF80	Fe_2_O_3_ (80 ppm)	837	3.21	4	41.96	55
	4d	TF120	Fe_2_O_3_ (120 ppm)	830	3.01	3	42.97	47.4
Ethanol [70]	5a	DF90E10	Absent	821.5	2.7	-	41.7	52.44
	5b	DF90E10	Al_2_O_3_ (100 ppm)	821.6	2.8	-	42.5	53.68
	5c	DF90E10	TiO_2_ (100 ppm)	821.6	2.8	-	42.3	53.24
Ethanol [71]	6a	TF	Absent	852	3.18	59	43.18	48.4
	6b	TF10	Al_2_O_3_ (10 ppm)	849	3.07	60	43.41	48.6
	6c	TF20	Al_2_O_3_ (20 ppm)	848	3.02	63	43.85	48.7
	6d	TF30	Al_2_O_3_ (30 ppm)	845	3.1	62	43.58	48.4
Ethanol [80]	7a	BDE	Absent	840.2	2.86	20	39.98	53
	7b	BDE	Al_2_O_3_ (25 ppm)	837.2	2.57	22	39.14	54
Methanol [75]	8a	M100	Absent	790	0.59	-	20.3	-
	8b	MCN25	CeO_2_ (25 ppm)	800	0.62	-	20.8	-
	8c	MCN100	CeO_2_ (100 ppm)	810	0.66	-	22.1	-
Isopropanol, Butanol [79]	9a	B20	Absent	847 *	3.70	-	43	42
	9b	D80SBD15E4S1	Al_2_O_3_ (100 mg/L)	840 *	3.37	-	42.59	52
Butanol [72]	10a	J50D10Bu	Absent	848 *	4.49	-	44.99	52.5
	10b	J50D10Bu25TiO_2_	TiO_2_ (25 mg/L)	849 *	4.51	-	45.11	53.5
	10c	J50D10Bu50TiO_2_	TiO_2_ (50 mg/L)	849 *	4.55	-	45.14	54.5
Butanol [81]	11a	B20But10	Absent	840.1	2.62	46.75	39.96	-
	11b	B20But10	TiO_2_ (0.01% by mass)	840.2	2.63	45	39.84	-
Ethanol [65]	12a	B2	Absent	820.7	2.31	-	42.66	-
	12b	B2E2C20	Carbon nanotubes (20 ppm)	821.8	2.39	-	42.23	-
	12c	B2E2C60	Carbon nanotubes (60 ppm)	821.8	2.38	-	42.27	-
	12d	B2E2C100	Carbon nanotubes (100 ppm)	821.9	2.39	-	42.23	-
	12e	B2E4C20	Carbon nanotubes (20 ppm)	820.7	2.31	-	42.66	-
	12f	B2E4C60	Carbon nanotubes (60 ppm)	820.8	2.31	-	42.68	-
	12g	B2E4C100	Carbon nanotubes (100 ppm)	820.9	2.31	-	42.62	-
	12h	B2E6C20	Carbon nanotubes (20 ppm)	819.6	2.24	-	43.11	-
	12i	B2E6C60	Carbon nanotubes (40 ppm)	819.7	2.24	-	43.13	-
	12j	B2E6C100	Carbon nanotubes (100 ppm)	819.9	2.25	-	43.03	-
Ethanol [78]	13a	B10	Absent	835	3.33	70	-	-
	13b	B10E2GQD30	Graphene quantum dot (30 ppm)	834	3.11	<28	-	-
	13c	B10E4GQD30	Graphene quantum dot (30 ppm)	834	2.99	<28	-	-
	13d	B10E6GQD30	Graphene quantum dot (30 ppm)	834	2.94	<28	-	-
	13e	B10E8GQD30	Graphene quantum dot (30 ppm)	834	2.83	<28	-	-
Heptanol [68]	14a	H20D	Absent	839.5 *	3.34	-	34.65	48.5
	14b	H40D	Absent	838.1 *	3.33	-	43.11	45.5
	14c	H20DMWCNT	Multi-walled carbon nanotubes (50 mg/L)	842.2 *	3.16	-	44.79	51.5
	14d	H20DGNP	Graphene nanoplatelets (50 mg/L)	842.1 *	3.11	-	44.79	50.5
	14e	H20DGO	Graphene oxide (50 mg/L)	842.3 *	3.12	-	44.80	51
	14f	H40DMWCNT	Multi-walled carbon nanotubes (50 mg/L)	841 *	3.16	-	43.60	49.5
	14g	H40DGNP	Graphene nanoplatelets (50 mg/L)	840.5 *	3.13	-	43.59	50
	14h	H40DGO	Graphene oxide (50 mg/L)	840.7 *	3.13	-	43.60	50.5
Butanol [77]	15a	JME40B	Absent	849.9 *	3.73	-	37.53	43.53
	15b	JME40B50GO	Graphene oxide (50 mg/L)	851.0 *	3.65	-	37.55	48.10
	15c	JME40BGNPs	Graphene nanoplatelets (50 mg/L)	851.1 *	3.68	-	37.56	47.95
	15d	JME40BMWCNTs	Multi-walled nanocarbon nanotubes (50 mg/L)	851.1 *	3.69	-	37.56	47.98

* Specific gravity.

**Table 2:** Summary of nanoparticles’ effect on fuel properties of vegetable oil/pure bio-oil-based fuels.

Vegetable Oil	Case #	Fuel	Nanoparticle (Dosage)	Density (kgm^−3^)	Viscosity (mm^2^/s)	Flash Point (°C)	Calorific Value (MJ/kg)	Cetane Number
Polanga seed oil [91]	1a	Neat polanga	Absent	937.4 *	57.8	-	-	-
	1b	Diesel + polanga	Fe_2_O_3_ (100 ppm)	835.3 *	3.49	-	44.08	-
	1c	Diesel + polanga	Fe_2_O_3_ (200 ppm)	837.3 *	3.62	-	44.03	-
	1d	Diesel + polanga	Fe_2_O_3_ (300 ppm)	837.5 *	3.39	-	44.00	-
Tyre oil ** [84]	2a	B10	Absent	820	6.59	49	42.90	-
	2b	B10D85	CeO_2_ (50 ppm)	822	6.65	50	42.94	-
	2c	B10D80	CeO_2_ (100 ppm)	824	6.72	51	42.98	-
Lemongrass oil [85]	3a	LGO25	Absent	870 *	3.48	53	41.69	-
	3b	LGO25 + WE + CE	CeO_2_ (50 ppm)	910 *	4.16	58	41.06	-
Pyrolyzed biomass oil ** [88]	4a	PBO20	Absent	845	4.24	96	41.1	-
	4b	PB020	Al_2_O_3_ (50 ppm)	839	4.08	94	41.2	-
	4c	PBO40	Absent	862	4.86	108	39.5	-
	4d	PBO40	Al_2_O_3_ (100 ppm)	852	4.72	104	41.3	-
Lemon peel oil [86]	5a	LPO20	CeO_2_ (50 ppm)	856	2.43	44	41.20	-
	5b	LPO20	CeO_2_ (100 ppm)	856	2.56	40	42.44	-
	5c	LPO20	CNT (50 ppm)	856	2.38	42	42.11	-
	5d	LPO20	CNT (100 ppm)	856	2.64	44	41.88	-
Orange peel oil [86]	6a	OPO20	CeO_2_ (50 ppm)	858	2.54	46	42.48	-
	6b	OPO20	CeO_2_ (100 ppm)	858	2.80	42	42.32	-
	6c	OPO20	CNT (50 ppm)	858	2.72	44	42.41	-
	6d	OPO20	CNT (100 ppm)	858	3.01	43	42.17	-
Nerium olender [87]	7a	ENOB	Absent	906	4.67	74	35.8	-
	7b	NENOB	CeO_2_ (30 ppm)	916.4	4.99	67	36.2	-
Lemongrass oil [82]	8a	Neat LGO	Absent	905	4.60	55	37	48
	8b	LGO emulsion	Absent	906	4.67	74	35.8	46.3
	8c	LGO nano emulsion	CeO_2_ (30 ppm)	916.4	4.99	67	36.2	48.8
Hydrotreated vegetable oil [89]	9a	B7 + 10%HVO	Absent	828.5	2.73	59	-	55.2
	9b	B7 + 10%HVO	CeO_2_ (1:4000)	828.3	2.73	60	-	53.1
	9c	B7 + 10%HVO	Nano ferrocen (1:1000)	828.1	2.72	59	-	57.7
Tyre pyrolysis oil ** [90]	10a	JME90TPO10	Absent	868.7	6.39	-	9962.7 ***	-
	10b	JME90TPO10	CeO_2_ (100 ppm)	868.3	6.39	-	9537.5 ***	-
	10c	JME90TPO10	CNT (100 ppm)	872.6	5.25	-	9311.5 ***	-
	10d	JME80TPO20	Absent	874.1	6.36	-	10,001.43 ***	-
	10e	JME80TPO20	CeO_2_ (100 ppm)	873.5	6.40	-	9630.2 ***	-
	10f	JME80TPO20	CNT (100 ppm)	878.1	5.35	-	9482.6 ***	-
	10g	JME70TPO30	Absent	880.4	6.48	-	10062 ***	-
	10h	JME70TPO30	CeO_2_ (100 ppm)	880.3	6.39	-	9726.8 ***	-
	10i	JME70TPO30	CNT (100 ppm)	881.8	5.29	-	9656.5 ***	-
Cymbopogon flexuosus biofuel [83]	11a	C20D80	Absent	843	3.21	49	42.19	-
	11b	C20D80	CeO_2_ (10 ppm)	844.1	3.28	47	42.14	-
	11c	C20D80	CeO_2_ (20 ppm)	844.5	3.31	46	41.88	-
	11d	C20D80	CeO_2_ (30 ppm)	844.9	3.37	45	41.62	-

* Specific gravity; ** not typical vegetable oil, but these neat oils share similar characteristics with vegetable oil; *** gross calorific value (cal/gm).

**Table 3:** Summary of nanoparticles’ effect on fuel properties of biodiesel-based fuels.

Biodiesel	Case #	Fuel	Nanoparticle (Dosage)	Density (kgm^−3^)	Viscosity (mm^2^/s)	Flash Point (°C)	Calorific Value (MJ/kg)	Cetane Number
Jatropha [92]	1a	JB20D	Absent	847.1 *	4.06	-	45.43	52
	1b	JB20D	MWCNT (10 mg/L)	847.1 *	4.1	-	45.43	52.7
	1c	JB20D	MWCNT (20 mg/L)	847.1 *	4.19	-	45.45	53.5
	1d	JB20D	MWCNT (30 mg/L)	847.1 *	4.25	-	45.45	54.2
	1e	JB20D	MWCNT (40 mg/L)	847.1 *	4.31	-	45.46	55.4
	1f	JB20D	MWCNT (50 mg/L)	847.1 *	4.35	-	45.46	56
Canola biodiesel [101]	2a	Canola biodiesel	Absent	886.5	5.38	172	38.76	48
	2b	Canola emulsion	CeO_2_ (50 ppm)	906.8	17.2	185	33.54	38
Jatropha [80]	3a	BDE	Absent	840.2	2.86	20	39.98	53
	3b	BDE	Al_2_O_3_ (25 ppm)	837.2	2.57	22	39.14	54
Jojoba [94]	4a	JB20	Absent	845.36	3.59	71	41.93	-
	4b	JB20CN25	CuO (25 ppm)	858.15	3.68	66	41.22	-
	4c	JB20CN50	CuO (50 ppm)	864.56	3.76	64	41.43	-
	4d	JB20CN75	CuO (75 ppm)	871.17	3.87	63	41.66	-
Rice bran [99]	5a	B20	Absent	828	6.62	39	38.96	-
	5b	B20	CeO_2_ (50 ppm)	830	6.16	35	39.44	-
	5c	B20	CeO_2_ (100 ppm)	826	5.96	45	39.25	-
Madhuca Indica [95]	6a	B100	Absent	889	5.21	173	40.30	-
	6b	B10A0.2	Al_2_O_3_ (0.2 gm)	848	4.38	65	41.78	-
	6c	B10A0.4	Al_2_O_3_ (0.4 gm)	853	4.35	63	41.82	-
	6d	B20A0.2	Al_2_O_3_ (0.2 gm)	858	4.49	59	41.91	-
	6e	B20A0.4	Al_2_O_3_ (0.4 ppm)	862	4.42	56	41.92	-
Palm oil [93]	7a	B100	Absent	860	4.61	-	38.6	62.5
	7b	B30C100	MWCNT (100 ppm)	852	5.12	-	40.3	52.2
Waste cooking oil [98]	8a	B5W3	Absent	-	3.6	78	44.35	-
	8b	B5W5	Absent	-	3.57	76	42.84	-
	8c	B5W7	Absent	-	3.92	74	42.49	-
	8d	B5W3_m_	CeO_2_ (90 ppm)	-	3.82	80	43.48	-
	8e	B5W5_m_	CeO_2_ (90 ppm)	-	3.82	78	42.73	-
	8f	B5W7_m_	CeO_2_ (90 ppm)	-	3.88	77	42.38	-
Neem oil [102]	9a	NBD	Absent	830	4.1	-	38.96	53
	9b	NBDCNT 50	Carbon nanotubes (50 ppm)	820	3.8	-	39.15	54
	9c	NBDCNT100	Carbon nanotubes (100 ppm)	810	3.5	-	39.56	55
Canola oil [96]	10a	B20	Absent	915	4.8	-	-	42
	10b	B20	TiO_2_ (300 ppm)	840	3.4	-	-	56
Kapok oil [103]	11a	B100	Absent	931	4.2	170	38	48
	11b	B20	Cobalt chromite (50 ppm)	845	3.8	145	39	49
Used cooking oil [104]	12a	B20	Absent	843.2	3.19	76	43.33	52.5
	12b	B20	MWCNT (25 ppm)	843.9	3.15	74	43.37	52.9
	12c	B20	MWCNT (50 ppm)	845.2	3.09	71	43.4	53.4
	12d	B20	MWCNT (75 ppm)	846.9	2.97	69	43.45	54.1
	12e	B20	MWCNT (100 ppm)	848.1	2.95	67	43.62	55.3
Garcinia gummi-gutta [97]	13a	B20	Absent	863	4.51	90.7	40.81	50.7
	13b	B20	TiO_2_ (25 ppm)	864	4.39	96.8	41.06	51.62
	13c	B20	CeO_2_ (25 ppm)	863	4.54	90.2	40.68	50.85
	13d	B20	ZrO_2_ (25 ppm)	866	4.51	93.1	41.31	50.91
Karanja oil/waste cooking oil [105]	14a	KBD20	Graphene oxide (60 ppm)	839	3.66	80	41.82	-
	14b	KBD20	Graphene nanoplatelets (60 ppm)	837	3.65	81	41.8	-
	14c	WBD20	Graphene oxide (60 ppm)	838	3.57	79	41.7	-
	14d	WBD20	Graphene nanoplatelets (60 ppm)	837	3.56	81	41.7	-
	14e	KBD20	Absent	836	3.65	81	41.8	-
	14f	WBD20	Absent	836.6	3.55	80	41.7	-
Orange peel oil [106]	15a	OOME	Absent	850.7	4.83	94	38.1	47
	15b	OOMET50	TiO_2_ (50 ppm)	856.5	5.17	96	35.98	50
	15c	OOMET100	TiO_2_ (100 ppm)	861.3	5.42	99	36.1	53
Waste frying oil [107]	16a	WFOME	Absent	898	4.21	160	43.85	-
	16b	WFOME	MWCNT (25 ppm)	830	4.75	57	43.73	-
	16c	WFOME	MWCNT (50 ppm)	831.1	4.45	65	43.93	-
Camelina oil [108]	17a	B20	Absent	836	5.67	-	44.09	-
	17b	B20G60	Graphene oxide (60 ppm)	832	5.53	-	44.49	-
Honge oil [109]	18a	HOME	Absent	-	5.6	170	36.02	-
	18b	HOME25CNT	MWCNT (25 ppm)	-	5.7	166	34.56	-
	18c	HOME50CNT		-	5.8	164	35.1	-
Sardine oil [110]	19a	SOME	Absent	890	4.5	58	37.41	45
	19b	SOME	CeO_2_ (25 ppm)	894	5.6	191	43.37	56
*Calophyllum inophyllum* [111]	20a	CIB20	Absent	843.3	3.56	69	40.92	53.85
	20b	CIB20ANP40	Al_2_O_3_ (40 ppm)	858	3.64	64	41.44	54.58

* Specific gravity.

**Table 4:** Summary of the most recent experiments on different nanoparticles addition in alcohol fuels used in CI engines.

Type of NPs Used	Alcohol Based Fuel	Blends	Size of NPs/NPs Concentration	Engine Sp.	Combustion		Performance		Gaseous Emission			Observation	Reference
					HRR	ICP	BFSC	BTE	CO	HC	NO_x_		
**Al_2_O_3_**	Ethanol	DF90E10 + A100, DF90E10 and DF	48 nm/100 ppm	Lombardini 15 LD 350, CI, CR20.3:1, RP7.5HP, RS3600 rpm, IP 207 bar.	---	↓ with DF90E10 + A100compared to DF	↓ by 2.25% with DF90E10 + A100 compared to DF	↑ by 3.48% with DF90E10 + A100 compared to DF	↓ by 25% with DF90E10 + A100 compared to DF	↓ by 30.15% with DF90E10 + A100 compared to DF	↓ by 3.02% with DF90E10 + A100 compared to DF	NPs-doped to DF90E10 acted as an oxygen-donating catalyst and ensured more oxygen atoms which in turn increase complete combustion.	[70]
	Ethanol	TF (TF + 10, TF + 20 and TF + 30) and DF	28 nm-30 nm/10 ppm, 20 ppm and 30 ppm	Kirloskar TAF 1, 1C, 4S, CI, CR17.5:1, RP4.4 kW, RS1500 rpm, IT 23° bTDC, IP 200 bar.	↑ highly with DF100 compared to other fuels	↓ by 2.33% with TF + 20 compared to DF	BSEC ↓ by 4.93% with TF + 20 compared to TF and DF	↑ by 7.8% with TF + 20 compared to TF and DF	↓ by 11.25% with TF + 20 compared to TF and DF	↓ by 5.63% with TF + 20 compared to TF and DF	↓ by 9.39% with TF + 20 compared to TF	TF + 20 was found to produce excellent combustion and emission behaviour. Al_2_O_3_ additives help to improve the catalytic combustion and shortened ID which in turn led to better air-fuel interaction.	[71]
	Methanol	MDFs (M10, M30, and M50); MD-NFs (M10A25, M10A50, M10A100, M30A25, M30A50, M30A100, M50A25, M50A50, and M50A100) and DF	30 nm/25 ppm, 50 ppm and 100 ppm	Kirloskar, 1C, 4S, CI, CR17.5:1, RP11.32 kW, RS2200 rpm, IT 20° bTDC, IP 18 MPa.	↓ with MDFs-NFs compared to DF and MDF at high load.	↑ by 30–50% with MD-NFs compared to MDFs and DF	---	---	↓ with MD-NFs highly compared to MDFs and DF	↓ with MD-NFs highly compared to MDFs and DF	↑ with MD-NFs significantly compared to MDFs and DF	NFs as additive leads to a definite reduction in CO and HC with an increase in NOx emissions. High LVH of methanol lead to reduction in temperature of in-cylinder charge In addition, MD-NFs lessen pre-combustion reactivity with increase in ID.	[123]
	Methanol	MDFs (M5 and M15); MD-NFs (M5A50, M5A100, M15A50 and M15A100) and DF	30 nm/50 ppm and 100 ppm	Kirloskar, 1C, 4S, WC, CI, CR17.5:1, RP11.32 kW, RS2200 rpm, IT 20° bTDC, IP 18 MPa.	Improved by 16.1% with ↓ of 6.9% in ID via 100 ppm	Improved by 2.5% with ↓ of 16% in CD via 100 ppm	↓ by ~3.7% with M5A100 and M15A100 compared to MDFs	↑ by ~3.6% with M5A100 and M15A100 compared to MDFs	↓ by 83.3% with MD-NFs compared to MDFs	↓ by ~40.9% with MD-NFs compared to MDFs	↑ slightly by 14.4% with MD-NFs compared to MDFs	Addition of NP in MDFs helps in improving the fuel cetane number which lead to improvement in HRR and ICR while reduction in ID and CD. Emissions were reduced compared to MDFs except NOx emission.	[121]
	Ethanol	D45EB10; D45EB10 + Al_2_O_3_ (D45EB10A50, D45EB10A75 and D45EB10A100)	30 nm/50 ppm, 75 ppm and 100 ppm	Kirloskar TVI, 1C, 4S, WC, CI, CR17.5:1, RP7 kW, RS1500 rpm, IT 23° bTDC, IP 220 kgf/cm^2^.	↑ highly with D45EB10A100 compared to D45EB10	↑ highly with D45EB10A100	---	---	↓ slightly by 0.02% with D45EB10A100 at 100% load.	---	↑ with an ↑ in Al_2_O_3_ rate and load.	Results showed that Al2O3 advances the initiation of combustion decreasing and shorten the ignition delay.	[124]
	Methanol and Ethanol	E.M.BioD.Al (5%Eth, 3%Meth, 86%BioD and 50 ppm)	20 nm/50 ppm	KIPOR KM186FA, 1C, 4S, AC, CI, CR19:1, RP5.7 kW, RS3000 rpms	Significantly improved	↑ highly with E.M.BioD.Al compared to other fuels	↓ with E.M.BioD.Al compared to BioD.	↑ by 6% with E.M.BioD.Al compared to BioD.	↓ by 12% with E.M.BioD.Al compared to BioD.	↓ slightly with E.M.BioD.Al compared to BioD.	↓ by 12.3% with E.M.BioD.Al compared to BioD.	Addition of Meth and NPs to the blend enhances the cetane number, which then results in an efficient combustion. The combustion of E.M.BioD.Al fuel provides more time for the oxidation of soot.	[115]
	Ethanol	JE20D; JE20D + Al_2_O_3_ (JE20D25A, JE20D50A, JE20D75A, JE20D100A) and DF	20 nm-50 nm/25 ppm, 50 ppm, 75 ppm and 100 ppm	HATZ-1B30-2, 1C, AC, CI, CR21.5:1, RP5.4 kW, RS3600 rpm, IT 20° bTDC, IP 18 MPa.	↑ highly with JE20D + Al_2_O_3_ compared to JE20D and DF	↑ highly with JE20D + Al_2_O_3_ compared to JE20D.	↓ by 17–25% with JE20D + Al_2_O_3_ compared to DF	↑ highly with JE20D + Al_2_O_3_ compared to JE20D	↓ by 20% with JE20D + Al_2_O_3_	↓ by 60% with JE20D + Al_2_O_3_	↓ by 30–50% with JE20D + Al_2_O_3_	The JE20D25A and JE20D75A blends improved the combustion process and resulted in lowered emissions compared to JE20D.	[119]
**CeO_2_**	Methanol	M10 and M30; MCN (M10C25, M10C100, M30C25 and M30C100) and DF	25 ppm and 100 ppm	Kirloskar, 1C, 4S, WC, CI, CR17.5:1, RP11.32 kW, RS2200 rpm, IT 20° bTDC, IP 18 MPa.	↑ by 7.9% slightly with MCN	↑ with MCN	↓ by 5.7–8.1% with MCN compared to M10 and M30	Improved by adding CeO_2_ to M10 and M30 with 5.2–108%	↓ by 79.8% with MCN compared to DF, M10 and M30.	↓ by 56.3% with MCN compared to DF, M10 and M30.	↓ by 70–90% with MCN compared to DF, M10 and M30.	Methanol and CeO_2_ NPs proved to be a promising technique for dual fuel in CI engines.	[75]
**CNTs**	Ethanol	D100, B2C20, B2C60, B2C100, B2E2C20, B2E2C60, B2E2C100, B2E4C20, B2E4C60, B2E4C100, B2E6C20, B2E6C60, and B2E6C100	4 nm–8 nm/20 ppm, 60 ppm and 100 ppm	DICOM 50.1 15/5, 1C, 4S, AC, DI, CI, RP9kW, RS3000 rpm.	---	---	↓ by 11.73% with an ↑ in CNTs NPs.	↑ by 13.97% with an ↑ in CNTs NPs.	↓ by ~5.47% with CNTs NPs	↓ by 31.72% with CNTs NPs	↑ by 12.22% with CNTs NPs.	B2E4C60 has the optimal performance and emissions. The negative effects of NPs on CI engine need to be investigated.	[65]
**Fe_2_O_3_**	Pentanol	TF (P10B20D70); TF40, TF80 and TF120	40 ppm, 80 ppm and 120 ppm	Kirloskar TVI, 1C, 4S, CRDI, CI, CR18.0:1, RP3.7 kW, RS3000 rpm, IP 250–500 kgf/cm^2^.	↑ with an ↑ in Fe_2_O_3_ NPs	Significantly improve with the presence of Fe_2_O_3_ NPs	↓ significantly by 4.93% with TF80 and TF120 compared to TF	↑ by 7.8% with TF120 compared to TF.	↓ significantly by 5.69% with TF120 compared to other fuels	↓ significantly by 11.24% with TF120 compared to other fuels	↓ significantly by 9.39% with TF120 compared to other fuels	Result showed that the chemical reactivity in combustion takes place very fast by decreasing the ID while completing the combustion rate.	[69]
**GQD**	Ethanol	BEGQD (B10E2GQD30, B10E4GQD30, B10E6GQD30 and B10E8GQD30) and DF	30 ppm	DICOM, 1C, 4S, AC, CI.	↓ by ~14.35% with BEGQD compared to DF.	---	---	---	↓ by ~29.54% with BEGQD compared to DF.	↓ by ~31.12% with BEGQD compared to DF.	↓ with DF compared to BEGQD.	GQD NPs improve the performance and emission behaviour of the CI engine fuelled with diesel-bioethanol-biodiesel blends.	[78]
**SiO_2_**	Methanol	MSN (M10); M10Si (M10Si25, M10Si50 and M10Si100) and DF	20 nm–30 nm/25 ppm, 50 ppm and 100 ppm	Kirloskar, 1C, 4S, CI, CR17.5:1, RP11.32 kW, RS2200 rpm, IT 20° bTDC, IP 18 MPa.	↑ by ~8.6% max with M10Si100.	---	↓ by 6.2% with an ↑ in SiO_2_ NPs.	↑ by 5.1% with an ↑ in SiO_2_ NPs.	↓ by 55.4% with SiO_2_ NPs.	↓ by 38.5% with SiO_2_ NPs.	↓ by 5.2% with SiO_2_ NPs.	Reductions in the ID and CD can be found in the cases of high NPs dosage.	[74]
**Nano-biochar**	Ethanol	DB2E2, DB4E4, DB6E6, DB8E8; DBE (with 25–125 ppm) and DF	25 ppm–125 ppm	CT-159, 1C, 4S, CI, CR 21:1	↓ by ~3% with DBE compared to DF.	---	---	---	↓ by ~0.03–0.015% with an ↑ in nano-biochar DBE.	↓ by ~28% with 125 ppm fuels compared to other fuels.	↓ by ~15% with 100 ppm compared to other fuels.	Nano-biochar NPs lead to an improved combustion efficiency and reduced pollutant emissions.	[113]
**TiO_2_**	Ethanol	DF90E10 + T100, DF90E10 and DF	48 nm/100 ppm	Lombardini 15 LD 350, CI, CR20.3:1, RP7.5HP, RS3600 rpm, IP 207 bar.	---	↓ with DF90E10 + T100 compared to DF	↓ by 1.26% with DF90E10 + T100 compared to DF	↑ by 2.94% with DF90E10 + T100 compared to DF	↓ by 21.43% with DF90E10 + T100 compared to DF	↓ by 26.47% with DF90E10 + T100 compared to DF	↓ by 1.57% with DF90E10 + T100 compared to DF	Result showed that biofuel worsen the emission, performance and combustion as compared to DF. The addition of TiO_2_-based NPs allows these worsened to drawback.	[70]
	Butanol	J50Bu10; JBu + TiO_2_ (J50Bu10T25 and J50Bu10T50) and DF	25 ppm and 50 ppm	HATZ-1B30-2, 1C, 4S, WC, VVA, CI, CR8.39:1, RS1000 rpm, IT 6° bTDC, IP 150 bar.	↑ with JBu + TiO_2_	↑ with JBu + TiO_2_	↑ by 15% highly with JBu + TiO_2_	↑ by 17% highly with JBu + TiO_2_	↓ by 30% significantly with JBu + TiO_2_	↓ by 50% significantly with JBu + TiO_2_	↑ with an ↑ in TiO_2_ NPs.	With TiO_2_ NPs, no negative effects were recorded on CI engine components.	[72]
	Butanol	B20 and B100; B20Bu20; B + TiO_2_ (B20 + TiO_2_ and B20Bu10 + TiO_2_) and DF	0.1689 g	3 LD 510, 1C, 4S, WC, CI, CR17.5:1, RP9 kW, RS3300 rpm, IP 190 bar.	---	↑ with B + TiO_2_	↓ by 27.73–28.37% with B + TiO_2_ compared to all other fuels.	↑ by 0.34–0.66% with B + TiO_2_ compared to other fuels.	↓ by 14–38% with B + TiO_2_ compared to all other fuels except B100.	↓ by 22.38–34.39% with B + TiO_2_ compared to all other fuels except B100.	↑ by 1.20–3.94% with B + TiO_2_ compared to other fuels.	n-butanol improved cold flow properties of fuel blends. Adding TiO_2_ in fuels has positively effect on engine performance.	[81]
	Butanol	B5 and B10; BTiO_2_ (B5T25, B5T50, B10T25 and B10T50) and DF	25 ppm and 50 ppm	Kirloskar, 1C, 4S, WC, CI, CR18:1, RP3.5 kW, RS1500 rpm, IT 25° bTDC.	---	↑ slightly with an ↑ in engine load.	↓ by 2.87–6.47% with all BTiO_2_ except B5T25 which ↑ by 7.91% compared toDF.	---	↓ by 22.34–36.17% with BTiO_2_ compared to DF.	---	↑ by 0.89–0.7.78% with B5T25, B10T25 and B10T50 while B5T50 ↓ by 2.69%.	Butanol and TiO_2_-based additives can be used as fuel without engine modification.	[122]
**ZnO**	Ethanol	D40B30E30; D40B30E30Z250; TFu (D40B30E30C6 and D40B30E30Z250C6) and DF	30 nm/250 ppm	Kirloskar TAF 1, 1C, 4S, CI, CR17.5:1, RP4.41 kW, RS1500 rpm, IT 23° bTDC, IP 200 bar.	↑ significantly with TFu compared to DF.	↑ by 8% and 13% with TFu compared to DF.	↑ by 14–39% with TFu compared to DF.	↓ by 9–21% with TFu compared to DF.	↓ by 62–92% with TFu compared to DF.	↑ by 21% with D40B30E30C6 and ↓ by 9% with D40B30E30Z250C6.	↓ by 16–35% with TFu.	Fuel solubility played a vital role for limiting the emissions effect while improving the combustion performance of the engine.	[73]

**Note:** All engines considered are research-based, solely for testing purposes; ↑ = increase; ↓ = decrease; AC = air-cooled; bDTC = before top dead centre; BSEC = brake specific energy consumption; C = cylinder; CD = combustion duration; CI = compressive ignition; CR = compression ratio; CRDI = common rail direct injection; DF = diesel fuel; DI = direct injection; GQD = graphene quantum dot; HRR: heat release rate; ICP = in-cylinder pressure; ID = ignition delay; IT = ignition timing; IP = injection pressure; LVH = latent vaporization heat; Max. = Maximum; MDF = methanol-diesel fuel; NPs = nanoparticles; RS = rated speed; RP = rated power; S = stroke; Sp. = specification; TF = ternary fuel; TFu = stable fuels; VVA = variable valve actuation; WC = water cooled.

**Table 5:** Summary of the most recent experiments on different nanoparticles addition in vegetable oil/pure bio-oil fuels used in CI engines.

Type of NPs Used	Vegetable Based Fuel	Blends	Size of NPs/NPs Concentration	Engine Sp.	Combustion		Performance		Gaseous Emission			Observation	Reference
					HRR	ICP	BFSC	BTE	CO	HC	NO_x_		
**Al_2_O_3_**	Mahua oil	MO; EMOA (EMOA25, EMOA50, EMOA75 and EMOA100) and DF	25 ppm, 50 ppm, 75 ppm and 100 ppm	Kirloskar AVI, 1C, 4S, WC, CI, CR16.5:1, RP3.7 kW, RS1500 rpm, IT 23° bTDC, IP 220 bars.	↑ with Al_2_O_3_ NPs.	↑ with Al_2_O_3_ NPs.	---	↑ by ~29.2% with EMOA100 compared to other fuels.	↓ by 87.4% with EMOA100 and ↓ by 24.2% with MO than DF.	↓ by 37.3% with EMOA100 compared to DF and MO.	↓ by 10% with EMOA than MO and ↓ by 51% with MO than DF.	The result of Al_2_O_3_ NP fuels showed better combustion reactivity due to high thermal conductivity than the latter.	[125]
	Waste plastic oil	WPO20; WPO20A10-I, WPO20A20-I, WPO20A10-II and WPO20A20-II	20 nm and 100 nm/10 ppm and 20 ppm	Kirloskar 240PE, 1C, 4S, DI, CR17.5:1, RP3.7 kW, RS1500 rpm, IT 23° bTDC, IP 200 bar.	↑ with WPO20A10-I and WPO20A20-I.	↑ with WPO20A10-I and WPO20A20-I.	↓ by 8–11% with WPO NP fuels.	↓ by 8–12.2% with WPO NP fuels.	↓ with NP fuels.	↓ with NP fuels.	↑ with an ↑ in load.	Low amount of NP had better emission and combustion performance compared to large amount.	[133]
	Lemon grass oil	B20; BA (B20A10, B20A20 and B20A30)	20 nm–30 nm/10 ppm, 20 ppm and 30 ppm	Kirloskar TV1, 1C, 4S, DI, CI, CR17.5:1, RP5.2 kW, RS1500 rpm, IT 23° bTDC, IP 220 bar	↑ by 20.4% with B20A20 compared to B20.	↑ by 4.75% with B20A20 compared to B20.	BSEC ↓ with an ↑ in loads and NPs rate.	↑ by 2.71% with B20A20 at full load.	↓ by ~15.15% with NPs fuels compared to B20.	↓ by ~5.98% with NPs fuels compared to B20.	↓ by ~2.2% with NPs fuels compared to B20.	HRR was better in case of Al_2_O_3_ NP and ICP is better with CeO_2_, as compared in literature.	[127]
**CeO_2_**	Tyre pyrolysis oil	B5, B10, B15 and B20; and B10D85C100	50 ppm and 100 ppm	Kirloskar TV1, 1C, 4S, DI, CR17.5:1, RP3.7 kW, RS1500 rpm, IT 23° bTDC, IP 200 bar.	↑ with CeO_2_ NP fuel	↓ slightly with B5 NP fuel compared to DF	---	Improved by 2.85% with B5D85C100	↓ by 13.33% with B5D85C100 compared to DF.	↓ by 3.0% with B5D85C100 compared to DF.	↑ slightly by 1.4% with B5D85C100.	Best result can be achieved with low blend rate and NP conc.	[137]
	Orange peel oil	OPO20; OPO20C50 and OPO20C100	32 nm/50 ppm and 100 ppm	Kirloskar TV1, 1C, 4S, DI, CI, CR17.5:1, RP5.2 kW, RS1500 rpm, IT 23° bTDC, IP 200 bar	↑ with an ↑ in loads and ICP.	Improved due to Ä.	↓ with an ↑ in loads and NPs rate.	↑ with an ↑ in loads and NPs rate.	↓ with and without NPs addition compared to DF due to Ã.	↓ with and without NPs addition compared to DF due to Ã.	↓ with an ↑ in CeO_2_ NPs due to Þ.	Better aromatic and good solubility of CeO_2_ in orange oil led to good combustion.	[86]
	Lemon grass oil	LGO; LGO emulsion, LGO nano-emulsion and DF.	10 nm–20 nm/20 ppm–80 ppm	Kirloskar TV1, 1C, 4S, WC, CI, CR17.5:1, RP5.2 kW, RS1500 rpm, IT 23° bTDC, IP 200 bar.	---	---	↓ with an ↑ in EP for all the fuels.	↑ by 31.25% with LGO nano-emulsion compared to other test fuels..	↓ by 15.21% with LGO nano-emulsion compared to other fuels.	↓ by 16.12% with LGO nano-emulsion compared to other fuels.	↑ with LGO emulsion compared to other fuels.	Results shows that CeO_2_ NPs help to reduce the CT of the A/F mixture, as the AE of N_2_ is higher.	[144]
	Ginger grass oil	G10C30, G20C30, G30C30 and G40C30; and DF	30 ppm	Kirloskar, 1C, 4S, CI, CR17.5:1, RP5.2 kW, RS1500 rpm, IT 23° bTDC, IP 200 bar.	---	---	↓ slightly with an ↑ in load.	↓ with an ↑ in load.	↑ with NP fuels	↓ with G40C30 compared to DF.	↓ with DF compared to NP fuels.	It was found that G10C30 had better result means of emission and performance.	[145]
	Nerium oleander	SFDF, NOB, ENOB and NENOB	15.01 nm/30 ppm	Kirloskar, 1C, 4S, WC, CI, CR17.5:1, RP5.2 kW, RS1500 rpm, IT 23° bTDC, IP 200 bar.	↓ with NP fuels compared to DF.	↓ with NP fuels	BSEC ↑ higher with NENOB compared to other fuels.	↓ with NOB compared to other fuels	↓ with NP fuels but ↑ with highest EP.	↓ significantly with NENOB compared to DF.	↓ slightly with NENOB compared to DF.	CI engine needs modification to have thermal efficiency. comparable to diesel.	[87]
	Lemon grass oil	LGO; LGO emulsion, LGO nano-emulsion and DF.	16.27 nm/30 ppm	Kirloskar, 1C, 4S, WC, CI, CR17.5:1, RP5.2 kW, RS1500 rpm, IT 27° bTDC, IP 200 bar.	↓ with NP fuels due to §	↓ with NP fuels due to Ÿ	BSEC ↑ with LGO nano-emulsion compared to other fuels.	↑ with LGO compared to other fuels.	↓ with LGO nano-emulsion but ↑ at highest EP.	↓ with LGO nano-emulsion compared to other fuels.	↑ with an ↑ in EP across all fuels.	Both ICP and HRR decreases with NP fuels. However, not all NPs contributes to the enhancement of engine combustion.	[82]
**Ce_0.7_Zr_0.3_O_2_**	Corn stalk pyrolysis bio-oil	CB10C50, CB15C50, CB20C50 and CB25C50; and DF	50 ppm	1C, 4S, WC, CI, CR17.0:1, RP13.2 kW, RS2200 rpm, IP 190 bar.	---	↓ with CBs fuels	↓ with CBs and ↑ with an ↑ in load.	↑ by CBs with an ↑ in EP.	↑ with CB25C50 and ↑ with an ↑ in load.	↑ with CB25C50 and ↑ with an ↑ in load.	↓ with CB25C50 compared to other fuels.	CBs exhibit lower CV compared to DF, which might consume more fuel to maintain the same EP with low comparability.	[138]
**MgO**	Municipal waste plastic oil	MPO20; MPO20M100 and DF	100 ppm	Kirloskar, 1C, 4S, DI, CI, CR17.5:1, RP3.5 kW, RS1500 rpm, IT 23° bTDC, IP 220 bar	↓ by 7.04% with MPO20M100 compared to DF and ↓ by 17.5% with MPO20 than DF.	↓ by 11.96% with MPO20M100 compared to DF and ↓ by 19.52% with MPO20 than DF.	↓ with MPO20M100 compared to MPO20.	↑ with MPO20M100 compared to MPO20.	↓ by 18.18% with MPO20M100 compared to MPO20.	↓ by 21.87% with MPO20M100 compared to DF.	↑ by 14.47% with MPO20M100 compared to DF.	Addition of NPs in plastic oil led to an increased max HRR compared to diesel fuel.	[139]
**MWCNT**	Lemon peel oil	LPO20; LPO20CNT50 and LPO20CNT100	10 nm/50 ppm and 100 ppm	Kirloskar TV1, 1C, 4S, DI, CI, CR17.5:1, RP5.2 kW, RS1500 rpm, IT 23° bTDC, IP 200 bar	↑ with an ↑ in loads and ICP.	Improved due to Ä.	↓ with an ↑ in loads and NPs rate.	↑ with an ↑ in loads and NPs rate.	↓ with and without NPs addition compared to DF due to Ã.	↓ with and without NPs addition compared to DF due to Ã.	↑ with an ↑ in MWCNT NPs due to ß.	Excess carbon led to improper mixing and thus increasing the HRR and ID period.	[86]
	Waste fishing net oil	WFNO; WM50 and DF	50 ppm	Kirloskar, 1C, 4S, WC, CI, CR17.5:1, RP3.5 kW, RS1500 rpm, IT 23° bTDC.	↑ with WFNO compared to other fuels.	↑ with WFNO compared to other fuels.	↓ by 3.87% with MWCNT fuel compared to WFNO.	↑ by 3.83% with MWCNT fuel compared to WFNO.	↓ by 25%	↓ by 9.09%	↓ by 5.25%	The engine results showed high efficiency with WFNO compared to MWCNT fuel.	[141]
**Rice husk**	Pine oil	B10 and B20; B10RH and B20RH; and DF	<100 nm/0.1% (I g/l)	Kirloskar TV1, 1C, 4S, DI, CI, CR17.5:1, RP5.2 kW, RS1500 rpm, IT 23° bTDC, IP 210 bar	---	---	↑ by 4.1–8.7% with BRH compared to DF.	↓ by 3.04% with RH NPs compared to DF.	↓ by 27.27% with B20RH compared to other fuels.	↓ by 19.64% with B20RH compared to other fuels.	↑ with an ↑ in RH NPs.	Result indicated a significant change in performance and emission with RH NP fuels.	[128]
**TiO_2_**	Linseed oil	LS100; PLS20; PLS50, PLS100, PLS150 and PLS200	25 nm–150 nm/50 ppm, 100 ppm, 150 ppm and 200 ppm	Kirloskar TV1, 1C, 4S, WC, CI, CR17.5:1, RP5.2 kW, RS1500 rpm, IT 23° bTDC, IP 200 bar.	---	---	↓ with an ↑ in load and TiO_2_ NPs conc.	↑ slightly by 8.11% with PLS200 compared to LS20.	↓ by 21.05% with PLS200 compared to LS20.	↓ by 33.82% with an ↑ in TiO_2_ NPs conc.	↑ by ~6.53% with an ↑ in TiO_2_ NPs conc.	It was observed that the linseed oil values of viscosity and density are almost equal to the diesel when pre-heated to 100 °C.	[129]
	Orange oil	OM; OMT50 and OMT100; DF	20 nm/50 ppm and 100 ppm	Kirloskar TV1, 1C, 4S, WC, CI, CR17.5:1, RP5.2 kW, RS1500 rpm, IT 23° bTDC, IP 200 bar.	↑ with TiO_2_ NPs fuels.	Improved with an ↑ in NPs.	↑ slightly with an ↑ in TiO_2_ NPs conc.	Improved for OMT50 and OMT100 by 1.6% and 3.0%, resp. compared to DF.	↓ significantly by 22.4% with OMT100 compared to DF.	↓ by 18.7% with OMT100 compared to DF.	↑ slightly by 7.2–10.4% with an ↑ in TiO_2_ NPs conc.	The presence of water molecules and NPs in the fuel lead to increase in ICP and ID period, which suddenly favours HRR.	[106]
	Plastic oil	CPD 2S 5W; PDO 2S 5W; CWT, PWT and DF	40 nm–50 nm/20 ppm, 40 ppm and 60 ppm	Kirloskar TAF1, 1C, 4S, AC, CI, CR17.5:1, RP4.4 kW, RS1500 rpm, IT 26° bTDC, IP 215 bar.	↑ by 4.12% PDO compared to CPD.	Improved with an ↑ in NPs.	↓ with an ↑ in load and TiO_2_ NPs conc.	↓ with an ↑ in load.	↓ with an ↑ in load and TiO_2_ NPs conc.	↓ with an ↑ in load and TiO_2_ NPs conc.	↓ with an ↑ in load and TiO_2_ NPs conc.	With TiO_2_ NP fuel, combustion and emission behaviour significantly improved.	[140]

**Note:** All engines considered are research-based, solely for testing purposes; ↑ = increase; ↓ = decrease; Þ = high heat absorbing capacity; Ã = better solubility and higher surface; Ä = fine atomization behaviour; AC = air-cooled; AE = activation energy; ß = longer ignition delay; bTDC = before top dead centre; BSEC = brake specific energy consumption; C = cylinder; CD = combustion duration; CI = compressive ignition; CR = compression ratio; Conc = concentration; CRDI = common rail direct injection; CWT = (CPD 2S 5W 20T, CPD 2S 5W 40T, CPD 2S 5W 60T); DF = diesel fuel; DI = direct injection; Eff = Efficiency; EP = engine power; HRR: heat release rate; ICP = in-cylinder pressure; ID = ignition delay; IT = ignition timing; IP = injection pressure; Max. = Maximum; Min = Minimum; MDF = methanol-diesel fuel; NPs = nanoparticles; PWT = (PDO 2S 5W 20T, PDO 2S 5W 40T, and PDO 2S 5W 60T); RS = rated speed; RP = rated power; S = stroke; Sp. = specification; WC = water cooled.

**Table 6:** Summary of the most recent experiments on different NPs addition in biodiesel fuels used in CI engines.

Type of NPs Used	Source of Biodiesel Fuel	Blends	Size of NPs/NPs Concentration	Engine Sp.	Combustion		Performance		Gaseous Emission			Observation	Reference
					HRR	ICP	BFSC	BTE	CO	HC	NOx		
**Al_2_O_3_**	Honge oil	HOME20, HOME2020, HOME2040, and HOME2060	10 nm/20 ppm, 40 ppm and 60 ppm	Kirloskar TV1, 1C, 4S, WC, CI, CR17.5:1, RP5.5 kW, RS1500 rpm, IT 23° bTDC, IP 205 bar.	↑ with HOME2040 compared to other fuels.	↑ with HOME2040 compared to other fuels.	↓ by 11.6% HOME2040 compared to HOME20.	↑ by 10.57% with HOME2040 compared to HOME20.	↓ significantly by 47.43% HOME2040 compared to HOME20.	↓ by 37.72% HOME2040 compared to HOME20.	↑ with an ↑ in Al_2_O_3_ NPs conc.	Min values of ID period and CD were achieved for HOME2040.	[165]
	Tamarind seed oil	TS20; TSA (TS20A30 and TS20A60) and DF	20 nm/30 ppm and 60 ppm	Kirloskar TVI, 1C, 4S, WC, DI, CI, CR17.5:1, RP5.2 kW, RS1500 rpm, IT 23° bTDC, IP 220 bars.	↑ with TS20 compared to TSA.	↑ with DF compared to TSA blend.	↑ with TSA fuels compared to TS20 blend.	↑ by 1.56% with TS20A60 compared to DF.	↓ by 56.6% with NPs fuels compared to DF.	↓ with an ↑ in Al_2_O_3_ NPs.	↑ higher with TS20A60 but lower than TS20.	Shorter ID period and better ignition properties of the NP additives results in enhanced premixed combustion.	[168]
	Pongamia oil methyl ester	B25; B25A (B25A50 and B25A100) and DF	50 ppm and 100 ppm	Kirloskar TVI, 1C, 4S, WC, DI, CI, CR16.5:1, RP3.7 kW, RS1500 rpm, IT 23° bTDC, IP 220 bars.	↑ with an ↑ in Al_2_O_3_ NPs rate.	↑ with an ↑ in Al_2_O_3_ NPs rate.	↓ highly with B25A100 compared to other fuels.	Improved with an ↑ in Al_2_O_3_ NPs.	↓ marginal with B25A compared to B25 and DF.	↓ marginal with B25A compared to B25 and DF.	↑ with an ↑ in Al_2_O_3_ NPs rate.	The current challenge is unburnt NPs from the exhaust of the diesel engine, which need to be investigated in order to safeguard the global environment.	[169]
**CeO_2_**	WCO	B20; B20C80-10, B20C80-30 and B20C80-80.	10 nm, 30 nm and 80 nm/80 ppm	Kirloskar TV1, 1C, 4S, WC, CI, CR17.5:1, RP5.2 kW, RS1500 rpm, IP 180 bar.	↑ slightly with B20C80-30	↑ by ~1.7% with B20C80-30 compared to other fuels.	↓ by 2.5% with B20C80-30 compared to DF.	↑ with CeO_2_ NPs conc.	↓ by 56% with B20C80-30 compared to DF.	↓ by 27% with B20C80-30 compared to DF.	↓ by 17% with B20C80-30.	30 nm-sized CeO_2_ NP was found the most effective in decreasing NOx compared to 10 nm and 80 nm-sized.	[159]
	Corn oil	CO10; CO10C25, CO10C50 and CO10C75	50 nm–70 nm/25 ppm, 50 ppm, and 75 ppm	Kirloskar TV1, 1C, 4S, WC, CI, CR17.5:1, RP5.2 kW, RS1500 rpm, IT 23° bTDC, IP 200 bar.	---	↑ with CeO_2_ NPs conc.	↓ with an ↑ in CeO_2_ NPs conc.	↑ with CO10C50 at max. eff. 34.8% and load.	↓ with CO10C50	↓ with CO10C50	↑ with an ↑ in load and CeO_2_ NPs conc.	High rate of ICP and temperature in combustion influenced the formation of NOx.	[164]
**Co(Al, Cr)_2_O_4_**	Kapok oil	SIT KC1-RET, SIT KC2-RET, SIT KC3-RET, SIT KC4-RET, SIT KC1-ADV, SIT KC2-ADV, SIT KC3-ADV, SIT KC4-ADV	50 ppm, 100 ppm, 150 ppm, and 200 ppm	Kirloskar SV1, 1C, 4S, WC, CR17.5:1, RP5.9 kW, RS1800 rpm, IT varies.	↑ by 5.09% with IT of 23CAD bTDC	↑ by 5.27% with IT of 23CAD bTDC	↓ by 21.23% with SIT KC4-ADV.	↑ by 7.2% with KC4-ADV than SIT.	↓ by 41.66% with SIT KC4-ADV	↓ by 37.86% with SIT KC4-ADV	↓ by 16.45% with SIT KC1-RET.	Better result can be obtained by IT retardation of the engine.	[103]
**FeO.Fe_2_O_3_**	Chicken fat oil	B10, B20 and B30; B10F50, B10F100, B10F150, B20F50, B20F100, B20F150, B30F50, B30F100 and B30F150	18.21 nm/50 ppm, 100 ppm, and 150 ppm	Kirloskar TV1, 1C, 4S, WC, CI, CR17.5:1, RP5.2 kW, RS1500 rpm, IT 23° bTDC, IP 200 bar.	---	---	↓ highly by 10.64% with B20F100 compared to B20.	Improved by 4.84% for B20F100 compared to B20.	↓ Max 56.66% with B30F100 compared to B30.	↓ by 22.72% with B30F100 compared to B30.	↓ by ~15.39% with FeO.Fe_2_O_3_ NP fuels.	No any side effects observed related to engine efficiency after four months. No carbon deposits were observed in the fuel tank and carburettor.	[172]
**MgO**	WCO	B’s (B10, B20 and B100); MgO NPs fuels (B10W30A, B20W30A and B100W30A) and DF	30 ppm	Kirloskar TVI, 1C, 4S, WC, CI, CR17.5:1, RP7 kW, RS1500 rpm, IT 23° bTDC, IP 220 kgf/cm^2^.	↓ with an ↑ in NPs.	↓ highly with MgO NPs fuels.	↑ with MgO NPs fuels compared to B’s.	↑ with MgO NPs fuels compared to B’s.	↓ with MgO NPs fuels compared to other fuels.	↓ with MgO NPs fuels compared to other fuels.	↑ with MgO NPs fuels and B’s compared to DF.	Result showed that MgO NPs can be used to improve the cold flow properties when used in CI engine.	[166]
**MW-CNTs**	WCO	B20; B20M20, B20M50, B20M75 and B20M100.	2nm–16 nm/20 ppm, 50 ppm, 75 ppm, and 100 ppm	Lombardini, 1C, 4S, WC, CI, CR17.5:1, RP5.77 kW, RS3000 rpm, IP 190 bar.	---	---	↓ by ~6.7% as the load ↑	Improved by ~7.4% with an ↑ NPs.	↓ with an ↑ in MWCNTs fuels.	↓ with an ↑ in MWCNTs fuels.	↑ significantly	Increasing engine load from significantly enhanced the emission behaviour,	[104]
	Jatropha	J20; J20C25, J20C50 and J20C100	20 nm–25 nm/25 ppm, 50 ppm, and 100 ppm	DEUTZ F1L511, 1C, 4S, AC, CI, CR17.5:1, RP5.77 kW, RS1500 rpm, IT 24° bTDC, IP 175 bar.	---	---	↓ with an ↑ in load.	↑ with an ↑ in load and NPs conc.	↓ with an ↑ in CNTs NP conc.	↓ with an ↑ in CNTs NP conc.	↓ partially with CNTs fuels at high loads..	J20C50 achieved improvement in engine performance and emissions reductions compared to other fuels.	[157]
**NiO**	Neem oil	NB25, NB25N25, NB25N50, NB25N75 and NB25N100	7 nm–10 nm/25 ppm, 50 ppm, 75 ppm, and 100 ppm	Rocket Engg, VCR, 1C, 4S, WC, CR17.5:1, RP4.8 kW, RS1500 rpm, IT 23–27° bTDC.	---	---	↓ with an ↑ in NP conc. at 27° bTDC.	↑ by 6.3% with NiO fuels.	↓ significantly by NiO fuels.	↓ significantly by NiO fuels.	↑ with NiO NP fuels by advanced fuel injection.	Advancing fuel IT with presence of NP improves the performance and reduces the engine emissions.	[162]
**SiO_2_**	Soybean	SB25; SB25S25, SB25S50 and SB25S75; DF	5 nm–20 nm/25 ppm, 50 ppm, and 75 ppm	Kirloskar VCR, 1C, 4S, CI, CR21.5:1, RP5HP, RS1800 rpm.	---	---	↓ with an ↑ in load.	↓ slightly with SiO_2_ fuels compared to DF.	↑ significantly by SiO_2_ NP fuels with an ↑ in load.	↑ slightly with SiO_2_ NP fuels compared to DF.	↑ significantly with an ↑ in load.	Not all NP and biofuels are considered as clean energy, but assessment needs to be done.	[173]
	WCO	B20; B20SiO_2_ and DF.	100 ppm	Lombardini 15 LD 350, 1C, 4S, WC, DI, CR20.3:1, RP7.5HP, RS3600 rpm, IP207 bar.	↑ with an ↑ in load for all fuels.	↑ with an ↑ in load for all fuels.	↓ by B10SiO_2_ with an ↑ in loads.	↑ with an ↑ in load for all fuels.	↓ slightly with B10SiO_2_ compared to DF.	↓ by 80.98% with B10SiO_2_ compared to DF.	↑ significantly with B10SiO_2_ compared to DF.	SiO_2_ -based NPs into biodiesel gives better results than biodiesel alone.	[163]
**TiO_2_**	Cottonseed oil	CSBD; CSBD50 and CSBD100; and DF	17 nm–28 nm/50 ppm, and 100 ppm	Kirloskar AV1, 1C, 4S, WC, CI, CR18.5:1, RP3.5 kW, RS1400 rpm, IT 23° bTDC, IP 200 bar.	---	---	↓ with an ↑ in load at all tested fuels.	↓ with CSBD and TiO_2_ NP fuels compared to DF.	↓ with an ↑ in TiO_2_ NP.	↓ by 14.7–16.2% with CSBD100 compared to DF.	↓ with an ↑ in TiO_2_ NP at all load conditions.	CSBD100 fuel exhibits 1.1–1.5%. improvement compared to CSBD.	[174]
	Palm oil	B0, B20; B20T60 and B20AOT60; DF	60 ppm	TECH-ED, 1C, 4S, WC, VRC, CR20:1, RP4 kW, RS1500 rpm.	---	---	↓ with B20AOT60 compared to B20.	↑ higher with B20AOT60 compared to other fuels.	↓ with B20AOT60	↓ with B20AOT60	↑ with B20 but much lesser with B20AOT60 compared to DF.	Better results were obtained with NP and AO as it acts as a deterrent in the fuel reaction.	[175]
**ZnO**	Grapeseed oil	GS; GSZ50 and GSZ100	36 nm/50 ppm and 100 ppm	Kirloskar TV1, 1C, 4S, CI, CR17.5:1, RP5.2 kW, RS1500 rpm, IT 27° bTDC, IP 200 bar.	↓ with GS compared to DF.	↓ with GS compared to DF.	↓ with DF compared to other fuels.	Max. ↑ was at GSZ100.	↓ with an ↑ in EP and NPs conc.	↓ by ~13% with GSZ100 compared to other fuels.	↑ with GS compared to DF.	The fuel consumption increases with ZnO NPs fuel conc.	[167]
**ZrO_2_**	*Garcinia gummi-gutta*	B100, B20 and B20Z25	25 ppm	Kirloskar TAF-1, 1C, 4S, AC, CR17.6:1, RP5.2 kW, RS1500 rpm, IT 23° bTDC.	↑ with B20	↑ with B20	↓ with B20Z25 compared to B100.	↓ with B20Z25 compared to DF.	↓ with B20Z25 compared to DF and B100.	↓ with NP fuel compared to DF.	↓ slightly by B20z25 with an ↑ in EP.	B20Z25 acquired better efficiency and minimal emissions than B20 and B100 fuels.	[97]

**Note:** All engines considered are research-based, solely for testing purposes; ↑ = increase; ↓ = decrease; AO = antioxidant; AC = air-cooled; bTDC = before top dead centre; BSEC = brake specific energy consumption; C = cylinder; CD = combustion duration; CI = compressive ignition; CR = compression ratio; Conc = concentration; CRDI = common rail direct injection; CV = calorific value; DF = diesel fuel; DI = direct injection; Eff = Efficiency; Engg = Engineering; EP = engine power; HRR: heat release rate; ICP = in-cylinder pressure; ID = ignition delay; IT = ignition timing; IP = injection pressure; Max. = Maximum; Min = Minimum; NPs = nanoparticles; RS = rated speed; RP = rated power; S = stroke; Sp. = specification; WC = water cooled; VCR = variable compression ratio; WCO = waste cooking oil.

**Table 7:** Observations from exergy-based studies regarding low carbon fuels and nano-additives in diesel engines.

Base Fuel	Nanoparticles	Remarks				Ref.
		Exergy	Economic	Environmental	Sustainability	
Diesel–ethanol (D90E10)	Al_2_O_3_ and TiO_2_ at 100 ppm	The exergy efficiency at all loads followed a decreasing trend in superiority in the order: D90E10Al_2_O_3_ > D90E10TiO_2_ > D100 > D90E10. Clearly the addition of nanoparticles to diesel–ethanol blends improved the exergy. The presence of NPs increased the heating values of the fuels. In addition to this, the combustion efficiency and exergy efficiency of the fuels improved by virtue of the catalytic effect, micro-explosions, oxygen buffering, and large surface area-to-volume ratio of the NPs which causes chemical reactions to accelerate and provides excellent thermal properties.	The presence of NPs to D90E10 led to a reduction in fuel consumption and the specific exergy of the base fuel was increased. This led to a decrease in the fuel cost flow rate. At all loads, the cost of crankshaft work per unit energy is $/GJ followed a decreasing trend in inferiority in the order: D90E10 > D90E10TiO_2_ > D90E10Al_2_O_3_ > D100. The exergoeconomic analysis thus favoured the nanofuels compared to diesel–ethanol blend.	The presence of NPs ensured higher exergy efficiencies and this led to the production of nanofuels with relatively lower environmental impact. At all loads, the environmental impact rate pr unit of break power followed a decreasing trend in inferiority in the order: D90E10 > D100 > D90E10TiO_2_ > D90E10Al_2_O_3_. Nanofuels have thus presented better exergoenvironmental feasibility compared to both pure diesel and diesel–ethanol blend.	The most sustainable test fuel according to their sustainability index was D90E10Al_2_O_3_. This is sequentially followed by D90E10TiO_2_, D100, and D90E10.	[34]
Diesel–canola oil biodiesel (C10)	TiO_2_ at 100 ppm and 3 different sizes (29 nm, 45 nm, and 200 nm)	The exergy loss and exergy destruction increase with increase in NP size. As NP size gets larger, there is a general reduction in surface area-to-volume ratio, catalytic activity while fuel consumption and exergy inlet rate increases. The aggregation of these events at larger NP sizes leads to a lower exergy efficiency. At all loads, the cumulative exergy efficiency followed a decreasing trend in superiority in the order: C10 + 28 nm TiO_2_ (81.60%) > C10 + 45 nm TiO_2_ (79.06%) > C10 + 200 nm TiO_2_ (77.37%) > D100 (74.98%) > C10 (71.50%).	Similarly, the presence of NPs led to a superior thermoeconomic results in nanofuels compared to pure diesel and its blend with biodiesel. The NPs improve energy and exergy efficiencies and this produced optimal thermoeconomic results. The best thermoeconomic results was obtained at the smallest NP size. However, an opposite trend is observed for the unit cost and specific exergy cost. In this context, neat diesel and C10 had an economic advantage over their NP-doped counterparts. Reducing the grain size of the NPs led to the production of a worst fuel from an economic point of view. The heating value of the base fuel increases in the presence of the NPs, causing an increment in specific exergy cost for the nanofuels. Despite this trend, it is worth noting that per their advantage in exergy efficiencies, nanofuels showed beneficial and superior exergoeconomic results against the base fuel.	-	At all engine loads, the highest sustainability index of the diesel engine was recorded for C10 + 28 nm TiO_2_ test fuel as a result of its superior exergy efficiencies in contrast to other test fuels. This is followed by C10 + 45 nm TiO_2_ > C10 + 200 nm TiO_2_ > D100 > C10 in a decreasing order of sustainability.	[39]
Diesel–biodiesel (B5)	Al_2_O_3_ at 50 and 100 ppm	Averagely, the presence of Al_2_O_3_ increased the exergy efficiency by 7.28% compared to B5. Similarly, the Addition of the NP to the base fuel reduced unaccounted losses by 31.8% on an average. Additionally, there was a slight change in exergy loss to the cooling water when the NP was used. It is worth noting that, increase in NP dosage led to superior exergy efficiencies and entropy generation results. The high surface area of the Al_2_O_3_ NP led high ignition qualities–shortening the combustion time. Al_2_O_3′_s high catalytic activity and surface area-to-volume ratio also ensure that the carbon activation temperature is lowered–leading to the promotion of fuel oxidation and complete combustion. Thermal properties thus increase and causes enhanced exergy efficiencies of the low carbon fuelled-diesel engines under the influence of the NPs.	-	-	-	[41]
Diesel–biodiesel (B5 and B10)	Hybrid nano catalysts additives comprising cerium oxide and molybdenum oxide on amide-functionalized MWCNTs at 30, 60, 90 ppm	The nano-additives provided sufficient oxygen to promote complete combustion and decrease the amount of exhaust air pollutants. The occurrence of these mechanisms in the cylinder by virtue of the inclusion of the nano-additives ensured that there is a decrease in the exergy rate of the exhaust gas and the heat transfer exergy rate of the diesel engine. Increasing the concentration of the nano-additives made this observation more obvious. Furthermore, the net exergy work rate of the diesel engine benefits from the presence of the nano-additives compared to the nano-additive-free blends. In addition to their oxygen buffering characteristics, the nano-additives exhibit nanocluster explosiveness which help the decomposition of sediments and deposits, and prevents their reformation. The absence of iron and carbon deposits reduces friction of the engine’s movable parts. These factors contributed to an increase in engine power and causes the net exergy work rate of the engine to increase. The net exergy work is directly proportional to the exergy efficiency. Hence, the exergy efficiency of the diesel engine increases with increase in the amount of nano-additives.	-	-	-	[42]
Diesel–biodiesel (B5) emulsified with water at concentrations of 3, 5, and 7 wt%. Tween 80 and Span 80 used as surfactants	Aqueous nano CeO_2_ at 0 and 90 ppm	The presence of water decreases the exergy efficiency of pure B5, but the situation is greatly improved with the addition of Aqueous nano CeO_2_. At all loads, B5 with 3 wt% water and 90 ppm of NP (B5W3_m_) showed the best exergy efficiency amongst all test blended fuels. Similar findings are witnessed for the thermal efficiency.	Despite the excellent results for exergy efficiency, the exergoeconomic analysis revealed that pure diesel was more favourable than B5W5m.	-	Due to its high exergy efficiency, B5W3m in the test engine had the most favourable sustainability index among all test blended fuels.	[38,178]
Diesel–waste cooking oil biodiesel (D90B10)	Al_2_O_3_, TiO_2_, SiO_2_ at 100 ppm	The exergy efficiency of pure diesel degrades after the addition of biodiesel. The trend is significantly reversed with the inclusion of the NPs. D90B10Al_2_O_3_ recorded the highest exergy efficiency. This is followed by D90B10SiO_2_ > D90B10TiO_2_ > D100 > D90B10. Similarly, the lowest and highest exergy destruction was observed D90B10Al_2_O_3_ and D90B10, respectively. In addition, the crankshaft work followed an increasing trend of superiority in the order D90B10 < D100 < D90B10TiO_2_ < D90B10SiO_2_ < D90B10Al_2_O_3_.	Adding NPs led to a decrease in fuel consumption–hence, at all load conditions, the highest and lowest cost flow rate was recorded by D100 and D90B10SiO_2_. In the same way, D90B10SiO_2_ recorded the lowest exhaust cost flow rate and loss cost flow rate, closely followed by D90B10Al_2_O_3_. However, for cost flow rate of crankshaft work, D90B10Al_2_O_3_ was the most economical ahead of D90B10SiO_2_. The exergo-economic factor for the nanofuels were superior than the base blend and pure diesel at all engine loads with D90B10SiO_2_ being the highest of all.	-	At each engine load, the depletion number of the diesel engine followed a decreasing trend in the order D90B10 < D100 < D90B10TiO_2_ < D90B10SiO_2_ < D90B10Al_2_O_3_. In addition, the sustainability index of the nanofuels were better than the base blend and pure diesel at all engine loads with D90B10Al_2_O_3_ being the highest of all.	[40]

## Data Availability

The data used in the bibliometric section of this review are available on the Web of Science Collection Database. All data used to support the findings of the remaining sections are included within the review.
